# CORO7 functions as a scaffold protein for the core kinase complex assembly of the Hippo pathway

**DOI:** 10.1074/jbc.RA120.013297

**Published:** 2020-11-23

**Authors:** Jina Park, Kyoungho Jun, Yujin Choi, Eunju Yoon, Wonho Kim, Yoon-Gu Jang, Jongkyeong Chung

**Affiliations:** 1 School of Biological Sciences, Seoul National University, Seoul, Republic of Korea; 2 Institute of Molecular Biology and Genetics, Seoul National University, Seoul, Republic of Korea

**Keywords:** CORO7, *Drosophila*, Hippo pathway, *pod1*, Src, CORO7, coronin 7, DSHB, Developmental Studies Hybridoma Bank, FBM, FERM-binding motif, GMR, glass multimer reporter, HA, hemagglutinin, HEK293T, human embryonic kidney 293T, Hpo, Hippo, LatB, latrunculin B, LATS1/2, large tumor suppressor kinase 1/2, MBD, MOB1-binding domain, MOB1, MOB kinase activator 1, MST1/2, mammalian sterile 20-like kinase 1/2, NF2, neurofibromatosis type II, PBST, PBS with 0.1% Triton X-100, SARAH, sav/rassf/hpo, SAV1, salvador family WW domain–containing protein 1, Sd, Scalloped, TBS, TEAD-binding sequence, TEAD, transcriptional enhanced associate domain, UAS, upstream activating sequence, YAP, yes-associated protein, Yki, Yorkie

## Abstract

The Hippo pathway controls organ size and tissue homeostasis through the regulation of cell proliferation and apoptosis. However, the exact molecular mechanisms underpinning Hippo pathway regulation are not fully understood. Here, we identify a new component of the Hippo pathway: coronin 7 (CORO7), a coronin protein family member that is involved in organization of the actin cytoskeleton. *pod1*, the *Drosophila* ortholog of *CORO7*, genetically interacts with key Hippo pathway genes in *Drosophila*. In mammalian cells, CORO7 is required for the activation of the Hippo pathway in response to cell–cell contact, serum deprivation, and cytoskeleton damage. CORO7 forms a complex with the core components of the pathway and functions as a scaffold for the Hippo core kinase complex. Collectively, these results demonstrate that CORO7 is a key scaffold controlling the Hippo pathway via modulating protein–protein interactions.

The Hippo signaling pathway plays a crucial role in the control of organ growth and tissue homeostasis (1). Various upstream signals, such as growth factors, mechanical stress, and nutrients, have been identified to regulate the Hippo pathway (1, 2, 3). This signaling pathway was initially discovered in *Drosophila* (4, 5) and was found to be evolutionarily conserved in higher eukaryotes (6). In mammalian cells, major signaling components of the Hippo pathway include two upstream kinases, mammalian sterile 20-like kinase 1/2 (MST1/2) and large tumor suppressor kinase 1/2 (LATS1/2), and two scaffold proteins, salvador family WW domain–containing protein 1 (SAV1) and MOB kinase activator 1 (MOB1), which form the core kinase complex inhibiting yes-associated protein (YAP) transcription factor, the primary effector of the pathway (7, 8). On unfavorable growth conditions, MST1/2 binds to and phosphorylates SAV1 (9, 10). MST1/2 also interacts with and phosphorylates MOB1, forming the SAV1–MST1/2–MOB1 complex (2, 11, 12). LATS1/2 is then recruited to this complex and phosphorylated by MST1/2 at its hydrophobic motif, although the recruitment mechanisms remain unclear (2, 13). It is critical to understand the scaffolding functions of MOB1 and SAV1, as they have crucial regulatory roles, such as promoting complex formation and subsequent phosphorylation events. Multiple mechanistic and structural studies on MOB1 have revealed its function as a scaffold facilitating sequential phosphorylation of the MST1/2–LATS1/2–YAP axis (12, 14, 15, 16, 17, 18). How SAV1 regulates protein interactions among the members of the core kinase complex, particularly through its binding to MST1/2, has also been demonstrated (19, 20, 21). In addition to its role as a scaffold for MST1/2, SAV1 recruits MST1/2 to the membrane where it activates LATS1/2, and the SAV1–neurofibromatosis type II (NF2) interaction is necessary for this regulation (22).

Phosphorylated LATS1/2 is released from MST1/2, and MOB1 induces autophosphorylation of LATS1/2 at its activation loop (23), which in turn facilitates LATS1/2 activation (24). Finally, LATS1/2 phosphorylates YAP, leading to its sequestration in the cytoplasm by 14-3-3 proteins and degradation by the β-transducin repeat-containing protein–E3 ligase complex (25, 26, 27). When the Hippo pathway is turned off, the unphosphorylated YAP translocates to the nucleus and functions as a coactivator of transcription factors. YAP mainly interacts with and regulates transcriptional enhanced associate domain (TEAD) 1/2/3/4 to induce transcription of a set of genes that promote cell proliferation and inhibit apoptosis (28, 29, 30).

Coronin 7 (CORO7) belongs to the WD40-repeat coronin protein family (31) and is unique as it has two WD40-repeat domains, whereas the other coronin proteins contain only one domain. In mammalian cells, CORO7 localizes to the cytosol and trans-Golgi network where it regulates organization of the actin cytoskeleton, Golgi morphology, and post-Golgi trafficking (32, 33). It has been reported that localization of CORO7 to the Golgi membrane requires CORO7 tyrosine phosphorylation by Src kinase (34). In addition, a study has shown that polyubiquitinated CORO7 is targeted to the trans-Golgi network and facilitates F-actin assembly (35). Despite the reports demonstrating a few roles of CORO7 regarding actin cytoskeleton and the Golgi apparatus, the association between CORO7 and the Hippo pathway has not been explored.

In this study, we identified CORO7 as a new regulator of the Hippo signaling pathway. Through protein interaction database analyses and genetic screens, *pod1*, the *Drosophila* ortholog of *CORO7*, was discovered to function in activating the Hippo pathway. In mammalian cells, CORO7 was shown to be necessary for the activation of the Hippo pathway by functioning as a scaffold protein for the Hippo core kinase complex. Finally, we suggested a possibility that Src tyrosine kinase regulates CORO7. Together, these data revealed that CORO7 is a player responsible for the assembly of the Hippo core kinase.

## Results

### pod1 genetically interacts with the Hippo signaling pathway genes

**Figure 1:**
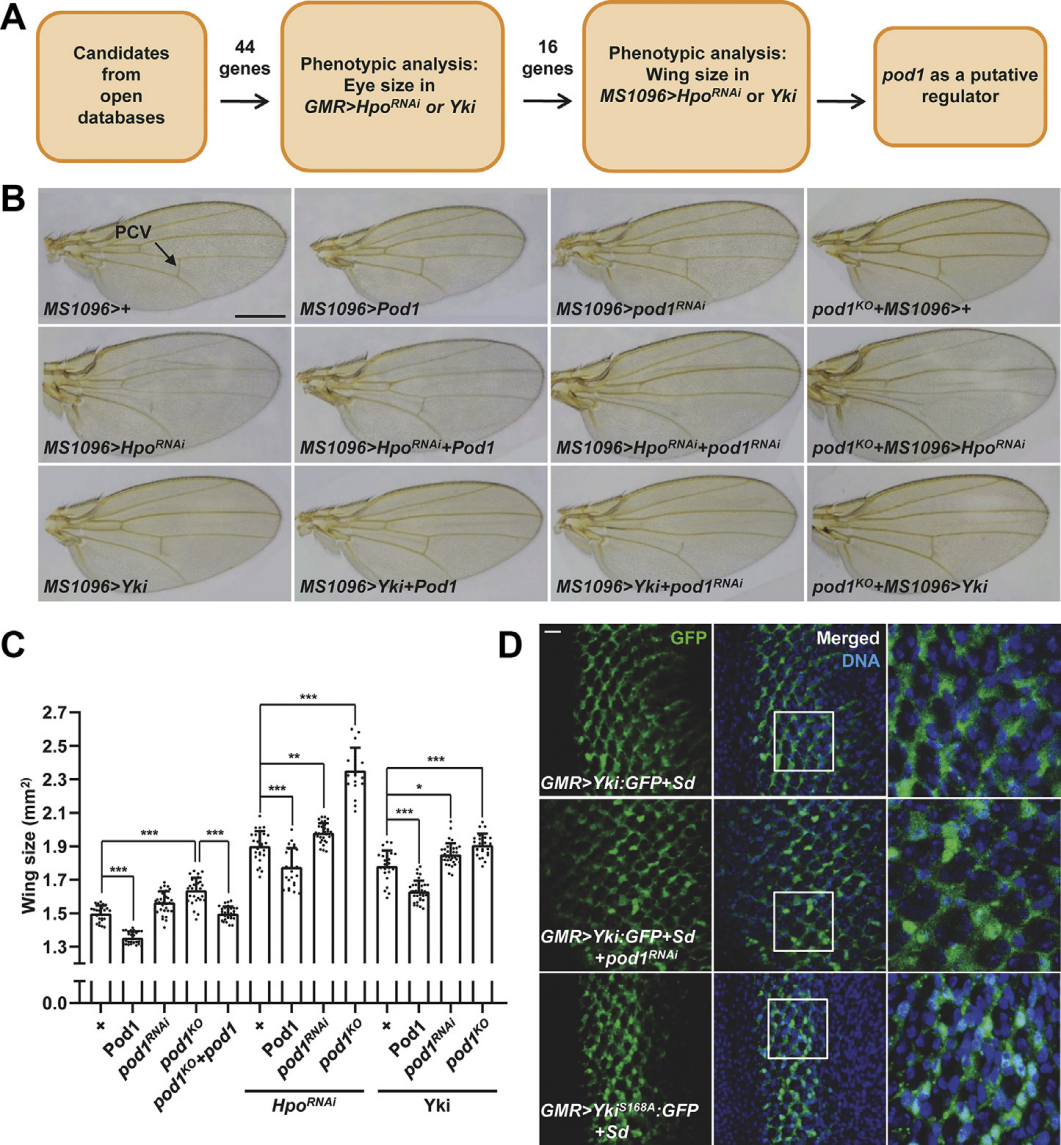
***Drosophila pod1* genetically interacts with Hippo pathway genes.***A,* schematic diagram of the genetic screen for Hippo pathway regulators. *B,* representative pictures of the wings of Pod1 overexpression (Pod1), *pod1* knockdown (*pod1*^*RNAi*^), *pod1* knockout (*pod1*^*KO*^) without or with *Hpo* knockdown (+*Hpo*^*RNAi*^) or Yki:GFP (+Yki) overexpression by *MS1096*-GAL4. The PCV is indicated. The scale bar represents 500 μm. *C,* statistical analysis of the wing size of each genotype in (*B*). The error bars represent ± S.D. from n > 20. One-way ANOVA and Tukey post-test were applied (∗*p* < 0.05; ∗∗*p* < 0.01; ∗∗∗*p* < 0.001). *D,* posterior regions of eye discs in *Drosophila* larvae. Yki:GFP, Yki^S168A^:GFP, Scalloped, and *pod1*^*RNAi*^ were expressed by *GMR-*GAL4 as indicated. The scale bar represents 10 μm. GMR, glass multimer reporter; PCV, posterior cross vein.

To identify new regulators of the Hippo signaling pathway, we listed *Drosophila* genes that have been reported to interact with the components of the pathway from multiple interactome databases, including BioGRID, BioPlex, STRING, and DroID (Table S1), and conducted a genetic screen by knocking down or overexpressing each gene. We used two GAL4 drivers, glass multimer reporter (GMR) and *MS1096*-GAL4, to express transgenes specifically in eyes and wings, respectively, because growth of these two organs during development is regulated by the Hippo pathway in *Drosophila* (4, 5, 20). The transgene was expressed alone or concomitantly with either *Hippo* (*Hpo*) knockdown or Yorkie (*Yki*) overexpression. *Hpo* and *Yki* are the *Drosophila* orthologs of *MST1/2* and *YAP*, respectively. Both *Hpo* knockdown and Yki overexpression mimic a situation where the Hippo pathway is inhibited, which leads to tissue growth, and our strategy was to find candidates that suppress the effects of such manipulations. Among the total 44 genes (Table S1) tested, *pod1* (*CG4352*) was chosen for further inspection (Fig. 1*A*).

Ectopic expression of Pod1 by *GMR*-GAL4 resulted in smaller eyes when expressed alone in the wild-type background or together with the Hippo pathway–inhibiting genetic manipulations mentioned previously (Fig. S1*A*), suggesting possible interactions between *pod1* and Hippo pathway genes. Consistently, Pod1 overexpression in wings led to significantly decreased wing sizes (Fig. 1, *B* and *C*). This was further confirmed by Pod1 overexpression experiments using another wing-specific GAL4 driver, *Hedgehog*-GAL4, which expresses the upstream activating sequence (UAS)-transgene in the posterior area of the *Drosophila* wing (Fig. S1, *B* and *C*). In addition, overexpression of Pod1 partially suppressed the phenotype caused by knockdown of *Hpo* or overexpression of Yki (Fig. 1, *B* and *C*). Although the knockdown of *pod1* alone did not result in significant size changes in eyes (Fig. S1*A*) and wings (Fig. 1, *B* and *C*), it enhanced the phenotype of *Hpo* knockdown or Yki overexpression in wings (Fig. S1*A* and Fig. 1, *B* and *C*). Furthermore, the posterior cross vein (*arrow* in Fig. 1*B*), which became faint on the suppression of the Hippo pathway, was clearly recovered when Pod1 was concurrently overexpressed. On the other hand, the double downregulation of *pod1* and the Hippo pathway induced a weaker vein phenotype (Fig. 1*B*). We speculated that if *pod1* interacts with the Hippo pathway, *pod1* knockout (KO) *Drosophila* would show a stronger phenotype compared with *pod1* knockdown mutants. To test this, we generated *pod1* KO *Drosophila* using the CRISPR/Cas9 system (Fig. S1*F*). As expected, complete removal of Pod1 protein resulted in larger wings compared with *pod1* knockdown under normal or the Hippo pathway–inhibiting conditions (Fig. 1, *B* and *C*). Furthermore, the increased wing-size phenotype was rescued by Pod1 expression in *pod1* KO mutants (Fig. 1*C*).

Because it has been reported that the Hippo pathway regulates tissue growth of the wing disc in *Drosophila* (36, 37, 38), we analyzed the imaginal disc morphology in Pod1 overexpression or *pod1* knockdown mutant larvae by staining with antibodies detecting disc-large and E-cadherin, which are apical markers of the tissue. Wild-type or Pod1-overexpressed *Drosophila* did not show any defect in imaginal disc structure, whereas *pod1* and/or *Hpo* knockdown induced the cellular overgrowth over the apical line of the imaginal disc (Fig. S1*D*). In addition, Pod1 overexpression rescued the overgrowth defect caused by *Hpo* knockdown, consistent with the observed phenotypes in wings (Fig. S1*D*).

To test whether *pod1* directly regulates the Hippo pathway, we investigated the localization of Yki in the *Drosophila* eye disc. A previous study has shown that a hyperactive form of Yki (Yki^S168A^) is localized in the nucleus when its binding partner, Scalloped (Sd), is overexpressed (39). Consistent with the report, we observed nuclear localization of Yki^S168A^ under conditions where Sd was overexpressed, whereas overexpressed wild-type Yki alone or Yki with Sd overexpression was localized in the cytoplasm (Fig. 1*D* and Fig. S1*E*). However, surprisingly, knockdown of *pod1* resulted in nuclear localization of wild-type Yki, indicating that Pod1 positively regulates the Hippo pathway and consequently inhibits Yki. Two additional experiments further confirmed Pod1 to be an upstream inhibitor of Yki*.* First, *yki* knockdown completely blocked the wing development of the *pod1* KO *Drosophila* (Fig. S1*G*). Second, *pod1* KO *Drosophila* showed elevated expression levels of *diap1*, a target gene of Yki, which was rescued by *heat shock*-GAL4–induced Pod1 overexpression (Fig. S1*H*). Together, these results demonstrated that Pod1 acts as an activator of the Hippo pathway in *Drosophila*.

### CORO7 is a positive regulator for the Hippo pathway in mammalian cells

**Figure 2:**
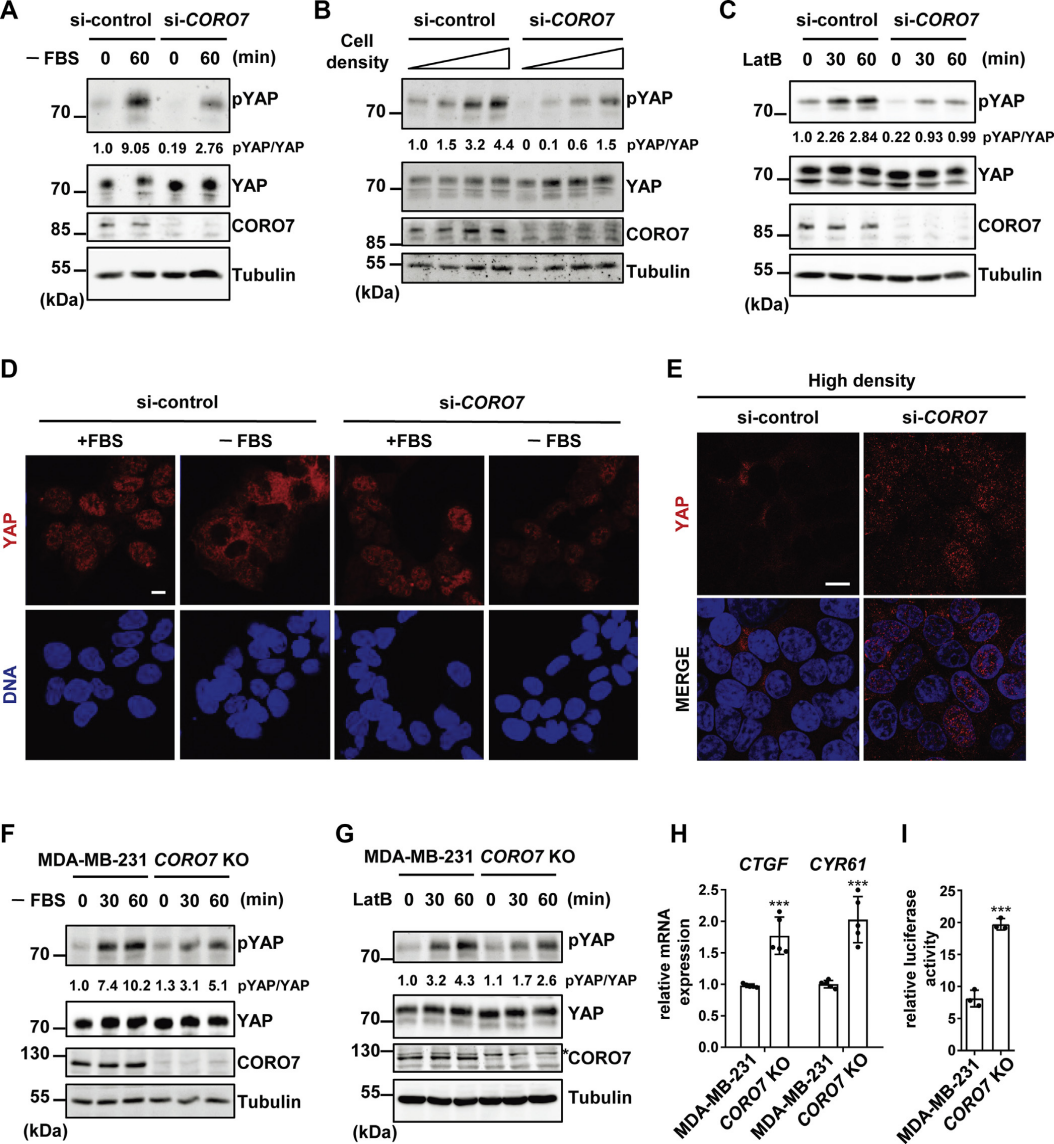
**CORO7 is critical for the activation of the Hippo pathway in mammalian cells.***A,* MDA-MB-231 cells were transfected with either nontargeting control siRNA (si-control) or siRNA targeting *CORO7* (si-*CORO7*) and were deprived of serum. The lysate samples were immunoblotted with anti-pYAP, anti-YAP, anti-CORO7, and antitubulin antibodies. *B,* equal numbers of MDA-MB-231 cells with (si-*CORO7*) or without (si-control) *CORO7* knockdown were seeded in 1.9, 3.8, 9.6, and 21.5 cm^2^ culture plates to achieve different cell densities 24  h before collecting, and the lysate samples were immunoblotted with the same antibodies as in (*A*). *C,* MDA-MB-231 cells were transfected with either nontargeting control siRNA (si-control) or siRNA targeting *CORO7* (si-*CORO7*) and were treated with LatB (0.25 μg/ml) to cause cytoskeleton damage. The cell lysate samples were immunoblotted with the same antibodies as in (*A*). Normalized signal intensities of pYAP bands relative to the total YAP protein levels were represented as numbers in (*A*–*C*). *D,* HEK293T cells were treated with either nontargeting control siRNA (si-control) or siRNA targeting *CORO7* (si-*CORO7*) and were serum starved for 1 h (FBS). The cells were immunostained with anti-YAP antibody (*red*). Nuclei of the cells were stained with Hoechst 33342 (*blue*). The scale bar represents 10 μm. *E,* HEK293T cells with (si-*CORO7*) or without (si-control) *CORO7* knockdown were cultured under a dense monolayer condition. The cells were immunostained with anti-YAP antibody. Nuclei of the cells were stained with Hoechst 33342 (*blue*). The scale bar represents 10 μm. *F* and *G*, wild-type (MDA-MB-231) or *CORO7*-lacking (*CORO7* KO) MDA-MB-231 cells were cultured under serum deprivation (*F*) or LatB treatment (0.25 μg/ml) (*G*). The lysate samples were immunoblotted with the same antibodies as in (*A*). Nonspecific band is indicated with an asterisk in (*G*). Normalized signal intensities of pYAP bands relative to the total YAP protein levels were represented as numbers. *H,* wild-type (MDA-MB-231) or *CORO7* KO MDA-MB-231 cells were cultured at high density as in (*B*) (lanes 4 and 8). mRNA expression levels of *CTGF* and *CYR61* were determined by quantitative real-time PCR and normalized by a control gene, *ACTB*. The error bars represent ±S.D. from five independent experiments. Student's two-tailed *t* test was applied (∗∗∗*p* < 0.001). *I,* wild-type (MDA-MB-231) or *CORO7* KO MDA-MB-231 cells were cultured at high density as in (*H*) and cotransfected with pRL-TK-encoding *Renilla* luciferase and 8× TBS-encoding firefly luciferase. About 24 h after the transfection, the cells were collected and subjected to dual-luciferase reporter assay (n = 3). Student's two-tailed *t* test was applied (∗∗∗*p* < 0.001). All immunoblots are representative of at least three independent experiments. CORO7, coronin 7; FBS, fetal bovine serum; KO, knockout; LatB, latrunculin B; YAP, yes-associated protein.

To identify whether CORO7 regulates the Hippo pathway in mammalian systems, we examined the role of CORO7 in the Hippo pathway when it is activated by various upstream signals. As previous research has shown that Ser127 of YAP is phosphorylated by LATS1/2 to induce the cytoplasmic sequestration of the protein on Hippo pathway activation (25), we tested the effect of *CORO7* depletion on this phosphorylation. As a result, *CORO7* knockdown significantly decreased the YAP phosphorylation induced by serum deprivation in MDA-MB-231 (Fig. 2*A* and Fig. S2*D*), HeLa (Fig. S2*A*), and human embryonic kidney 293T (HEK293T) cells (Fig. S2*B*). Consistently, cytoplasmic localization of YAP under this condition was also blocked by silencing *CORO7* (Fig. 2, *D* and Fig. S2*G*), indicating the necessity of *CORO7* in activating the Hippo pathway. Furthermore, *CORO7* knockdown impeded Hippo pathway activation induced by contact inhibition, as shown by reduced YAP Ser127 phosphorylation and its nuclear localization in densely cultured cells (Fig. 2, *B* and *E* and Fig. S2, *E* and *G*). Consistent with several studies suggesting that the actin cytoskeleton damage activates the Hippo pathway (40, 41, 42), YAP Ser127 phosphorylation was induced by treatment with latrunculin B (LatB), an inhibitor of the actin polymerization (Fig. 2*C*), or by cell detachment (Fig. S2*C*). The loss of *CORO7*, however, led to decreased Ser127 phosphorylation on LatB treatment or cell detachment (Fig. 2*C* and Fig. S2, *C* and *F*), suggesting that CORO7 also positively regulates the Hippo pathway in response to depolymerization of the actin cytoskeleton.

To further verify whether CORO7 is required for the Hippo signaling cascade, we generated MDA-MB-231 cells lacking *CORO7* using the CRISPR/Cas9 system. The deletion of *CORO7* was confirmed by immunoblotting with an anti-CORO7 antibody (Fig. S3*A*) and genomic DNA sequencing (Fig. S3*B*). Consistent with the results from the knockdown experiments, CORO7 KO cells showed decreased YAP Ser127 phosphorylation in response to serum deprivation (Fig. 2*F* and Fig. S3*E*) or LatB treatment (Fig. 2*G* and Fig. S3*F*). Because YAP phosphorylation by Hippo pathway activation results in the repression of YAP transcriptional activity, we next investigated whether *CORO7* depletion affects mRNA expressions of two YAP target genes, *CTGF* and *CYR61*. As expected, the expressions of both genes were increased in *CORO7* KO cells compared with wild-type cells (Fig. 2*H*). This result was also corroborated by a luciferase reporter assay. The activity of YAP was examined by the luciferase reporter transcription controlled by eight repeats of TEAD-binding sequence (8×TBS), as YAP is known to bind to TEADs to promote their transcriptional activity. We observed that YAP was significantly more active in *CORO7* KO cells compared with wild-type cells (Fig. 2*I*). Finally, we tested the possibility that loss of *CORO7* may disrupt the actin cytoskeleton, which can affect the Hippo pathway. The actin cytoskeleton was observed using phalloidin staining, and there were no discernable differences in actin structure between *CORO7* wild-type and KO cells (Fig. S3, *G* and *H*). Together, these data demonstrated that *CORO7* is necessary for the activation of the Hippo pathway under diverse conditions.

### CORO7 forms a complex with the components of the Hippo pathway

**Figure 3:**
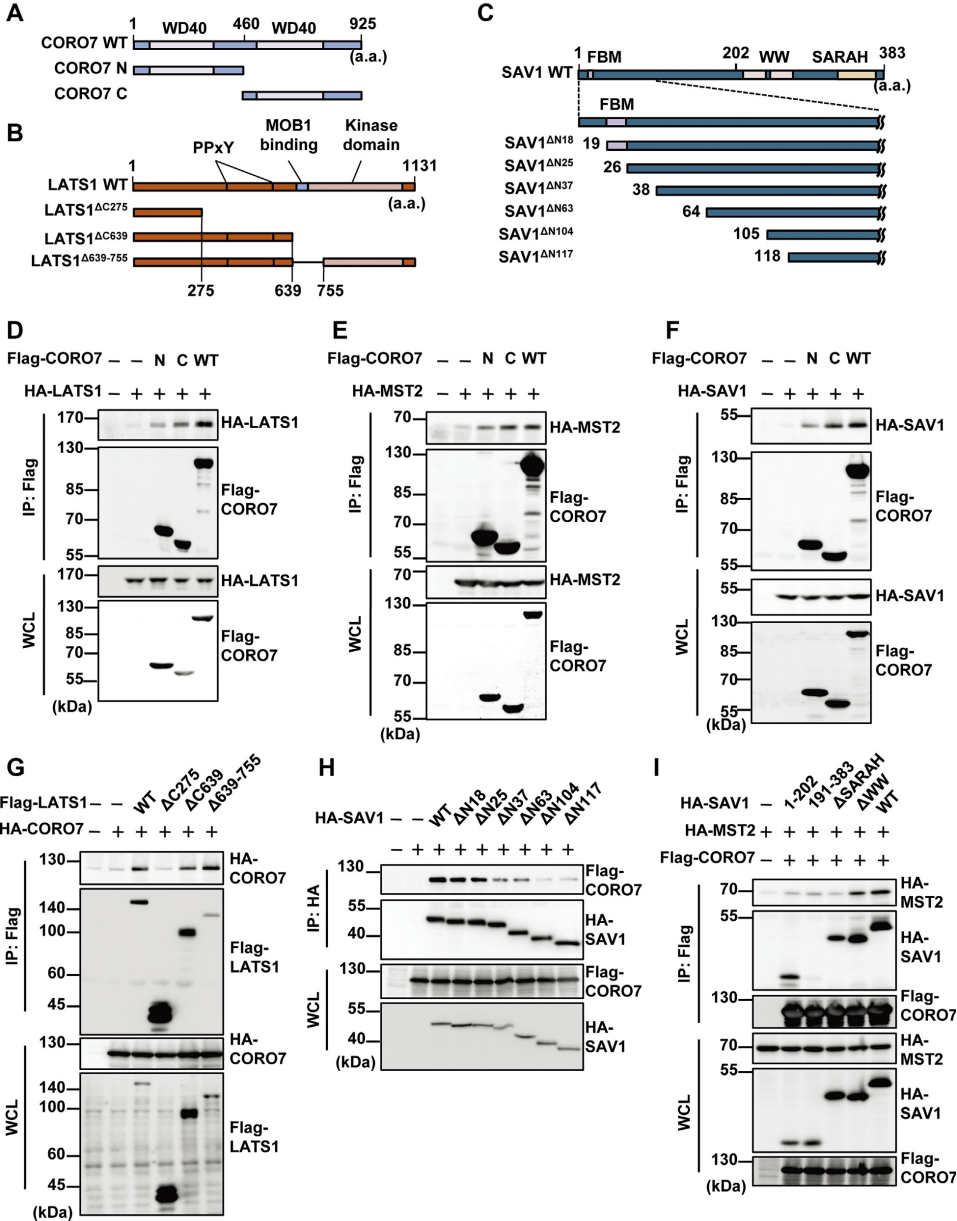
**CORO7 forms a complex with the components of the Hippo pathway.***A–C,* schematic representation of domains and truncated constructs of CORO7 (*A*), LATS1 (*B*), and SAV1 (*C*) used in coimmunoprecipitation assays. *D*–*F,* HA-LATS1 (*D*), HA-MST2 (*E*), or HA-SAV1 (*F*) was expressed together with wild-type (WT), N-terminal (N), or C-terminal (C) Flag-CORO7 in HEK293T cells. The lysates were immunoprecipitated by anti-Flag antibody and immunoblotted with anti-HA and anti-Flag antibodies. The whole cell lysate (WCL) samples were loaded for indicating the expression levels. *G,* HA-CORO7 was transfected with the Flag-tagged truncated forms of LATS1 in HEK293T cells, and the lysates were immunoprecipitated by anti-Flag antibody. Subsequently, they were immunoblotted with the same antibodies as in (*D*–*F*). The WCL samples were loaded for indicating the expression levels. *H,* Flag-CORO7 was transfected with the HA-tagged truncated forms of SAV1 in HEK293T cells. The lysates were immunoprecipitated by anti-HA antibody and immunoblotted with the same antibodies as in (*D*–*F*). The WCL samples were loaded for indicating the expression levels. *I,* HEK293T cells were transfected with HA-tagged truncated forms of SAV1, HA-MST2, and Flag-CORO7. The lysates were immunoprecipitated by anti-Flag antibody and immunoblotted with the same antibodies as in (*D*–*F*). The WCL samples were loaded for indicating the expression levels. Data information: Flag-tagged vector and HA-tagged vector were transfected as negative controls (−). All immunoblots are representative of at least three independent experiments. CORO7, coronin 7; FBM, FERM-binding motif; HA, hemagglutinin; LATS1, large tumor suppressor kinase 1/2; MOB1, MOB kinase activator 1; MST2, mammalian sterile 20-like kinase 2; SARAH, Sav/Rassf/Hpo; SAV1, salvador family WW domain–containing protein 1; WCL, whole cell lysate.

WD40-repeat domain plays essential roles in several signaling pathways as a scaffold, which facilitates protein–protein interaction (43). Because CORO7 has two highly conserved WD40-repeat domains, we tested whether it forms a complex with the core components of the Hippo pathway and found that CORO7 interacted with a subset of the components of the Hippo pathway, including LATS1, MST2, and SAV1 (Fig. 3, *D*–*F* and Fig. S4*C*) but not with MOB1 (Fig. S4*G*). We also observed that endogenous CORO7 coimmunoprecipitated with endogenous LATS1 (Fig. S4*A*) and SAV1 (Fig. S4*B*). To identify the region of CORO7 that is responsible for these interactions, we expressed the N-terminal (1-459 amino acids) or C-terminal half (460-925 amino acids) of CORO7 (Fig. 3*A*) and conducted coimmunoprecipitation assays. As a result, the C-terminal half showed comparable interactions with LATS1, MST2, and SAV1 to the full-length CORO7, whereas the N-terminal counterpart did not show significantly stronger interactions with the three binding partners compared with the vector control (Fig. 3, *D*–*F* and Fig. S4*C*).

To examine the interaction of CORO7 with LATS1 or SAV1 in detail, we further mapped the CORO7-binding regions in both LATS1 and SAV1. Based on reported structures of LATS1/2 and SAV1 (12, 44), we prepared truncated forms of them for coimmunoprecipitation assays (Fig. 3, *B* and *C*). For LATS1, we tested three kinds of truncated forms: (1) LATS1^ΔC275^ lacking the PPxY motifs for YAP binding, the MOB1-binding domain (MBD), and the kinase domain (2), LATS1^ΔC639^ containing the two PPxY motifs but not the MBD and kinase domain, and (3) LATS1^Δ639-755^ lacking only the MBD (Fig. 3*B*). For SAV1, we first used the N-terminal half (SAV1^1-202^) and C-terminal half (SAV1^191-383^) to show that the former is necessary for the interaction between SAV1 and CORO7 (Fig. S4*D*), and hence adopted six kinds of truncated SAV1 that lack different numbers of N-terminal amino acids (Fig. 3*C*). Of note, at the N terminus of SAV1 lies the 4.1/Ezrin/Radixin/Moesin-binding motif (FBM), which has been appreciated to be required for NF2 to interact with SAV1 via its 4.1/Ezrin/Radixin/Moesin domain (45).

Using the truncated forms of LATS1 and SAV1, we found that LATS^276-639^ is required for LATS1–CORO7 binding (Fig. 3*G*), whereas the N-terminal end of SAV1 is responsible for SAV1–CORO7 binding (Fig. 3*H*). We observed a clear decrease in interaction between SAV1 and CORO7 when truncated forms of SAV1 lacked their N-terminal 37 or 63 amino acids and further loss of interaction when 104 or 117 N-terminal amino acids were removed (Fig. 3*H*). In addition, the FBM of SAV1 was dispensable for the interaction between SAV1 and CORO7 (Fig. S4*E*).

It has been reported that MST1/2 binds to SAV1 through its sav/rassf/hpo (SARAH) domain and phosphorylates SAV1 (9, 10). As SAV1 is believed to act as a scaffold for MST1/2, we next examined whether the formation of the SAV1–MST1/2 complex is involved in the interaction of CORO7 and MST2. Indeed, a SARAH domain-lacking mutant form of MST2, which cannot bind to SAV1, was unable to interact with CORO7 (Fig. S4*F*). Furthermore, coexpression of wild-type SAV1 enhanced the interaction between CORO7 and MST2 (Fig. 3*I*), suggesting that SAV1 may mediate the binding of MST2 to CORO7. We also investigated which domain of SAV1 is responsible for its function as a scaffold linking CORO7 and MST2. Coimmunoprecipitation experiments showed that the N-terminal half (SAV1^1-202^), which interacts with CORO7, and the SARAH domain, which binds to MST2, but not the WW domain of SAV1, are required to augment the interaction between CORO7 and MST2 (Fig. 3*I*).

### CORO7 is necessary for the formation of the Hippo core kinase complex

One of the most important regulatory mechanisms of the Hippo pathway is the sequential phosphorylation of the MST1/2-LATS1/2-YAP axis. It has been reported that SAV1 interacts with the PPxY motifs of LATS1/2 through its WW domains (46). A recent structural study, however, showed that the binding between SAV1 WW domains and a PPxY-containing peptide of LATS1 is much weaker than the binding between MOB1 and the MBD of LATS1 (12, 17). Furthermore, little is known about how SAV1–LATS1/2 binding is regulated, and there is no direct evidence for the formation of the MST1/2–SAV1–LATS1/2 ternary complex (12). In short, detailed information regarding how the Hippo core kinase complex is formed and regulated remains to be elucidated, and we hypothesized that CORO7 has a role in the formation of the complex. To test the idea, we performed coimmunoprecipitation assays to observe the association between LATS1 and MST2. As expected, LATS1 and MST2 were found to be in the same complex when both SAV1 and MOB1 were coexpressed (Fig. S5*A*). Notably, wild-type SAV1, but not SAV1^ΔN37^, enhanced the interaction of LATS1 and MST2 (Fig. S5*A*), suggesting that the N-terminal end of SAV1, which includes the CORO7-binding site, is required for the scaffold function of SAV1. Besides, although SAV1 has been reported to bind to LATS1/2 through its WW domain, the SAV1^ΔN37^ containing the WW domain failed to bind to LATS1 (Fig. S5*A*), indicating that the N-terminal end of SAV1 is also necessary for the interaction between SAV1 and LATS1.

**Figure 4:**
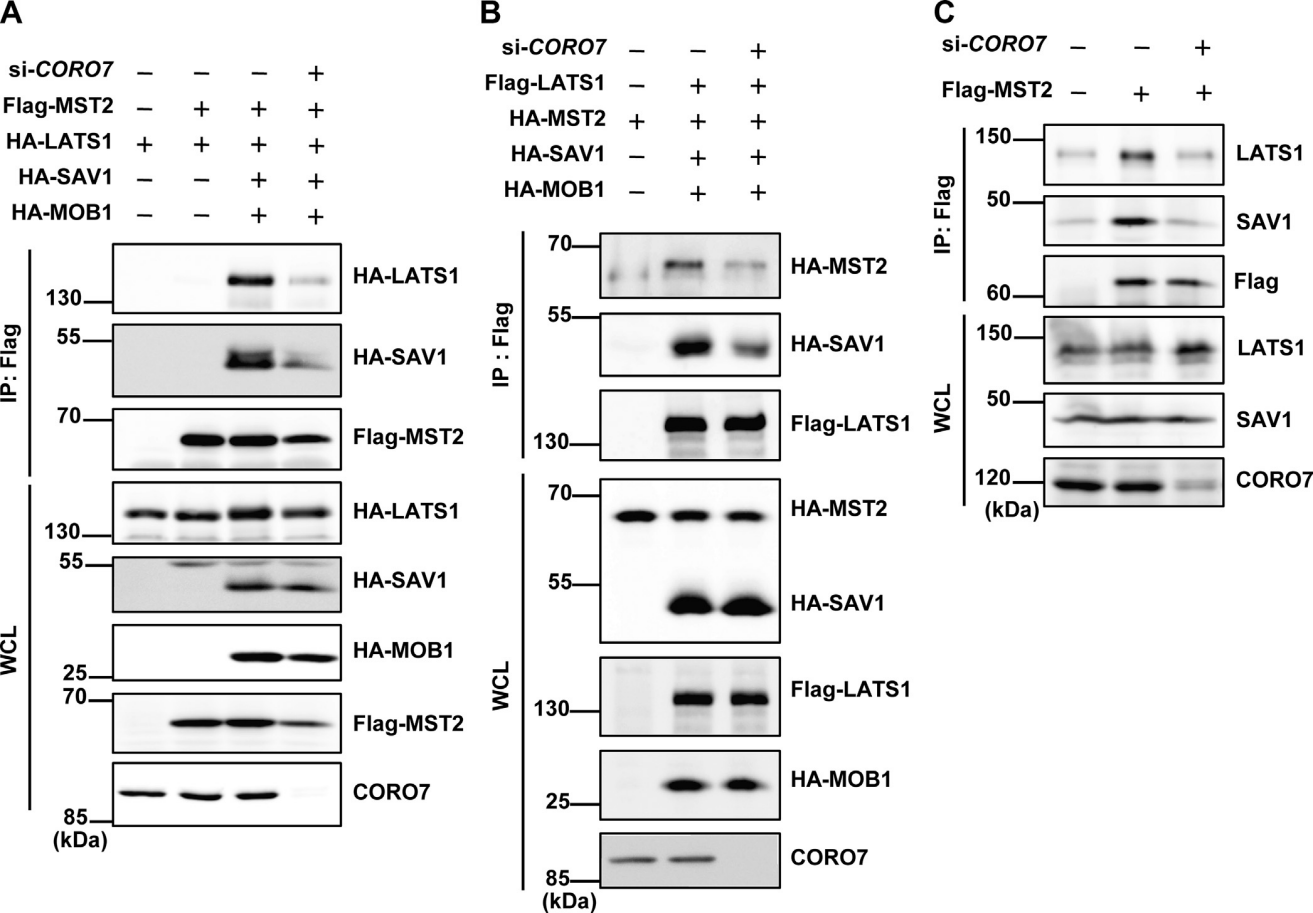
**CORO7 is necessary for the formation of the core Hippo kinase complex.***A,* Flag-MST2, HA-LATS1, HA-SAV1, and HA-MOB1 were transfected in HEK293T cells to observe the coimmunoprecipitation between Flag-MST2 and the rest. siRNA targeting *CORO7* was treated in the indicated lane (si-*CORO7*). The lysates were immunoprecipitated by anti-Flag antibody and immunoblotted with anti-HA and anti-Flag antibodies. The whole cell lysate (WCL) samples were loaded for indicating the expression levels, and CORO7 was immunoblotted to verify the efficiency of knockdown. *B,* flag-LATS1, HA-MST2, HA-SAV1, and HA-MOB1 were transfected in HEK293T cells to observe the coimmunoprecipitation between Flag-LATS1 and the rest. siRNA targeting *CORO7* was treated in the indicated lane (si-*CORO7*). The lysates were immunoprecipitated by anti-Flag antibody and immunoblotted with anti-HA and anti-Flag antibodies. The WCL samples were loaded for indicating the expression levels, and CORO7 was immunoblotted to verify the efficiency of knockdown. *C,* flag-MST2 was expressed in HEK293T cells, and *CORO7* was knocked down in the indicated lane (si-*CORO7*). Cells were deprived of FBS for 24 h, and the lysates were immunoprecipitated by anti-Flag antibody and immunoblotted with anti-LATS1, anti-SAV1, and anti-Flag antibodies. The WCL samples were loaded for indicating the expression levels, and CORO7 was immunoblotted to verify the efficiency of knockdown. All immunoblots are representative of at least three independent experiments. CORO7, coronin 7; HA, hemagglutinin; LATS1, large tumor suppressor kinase 1/2; MOB1, MOB kinase activator 1; MST2, mammalian sterile 20-like kinase 2; SAV1, salvador family WW domain–containing protein 1; WCL, whole cell lysate.

We next tested whether CORO7 is necessary for the interaction among the core components of the Hippo pathway. Despite coexpression of SAV1 and MOB1, knockdown of *CORO7* partially blocked the interaction between MST2 and LATS1 (Fig. 4*A* and *B* and Fig. S5, *B*–*D*). Furthermore, the silencing of *CORO7* decreased the degree of SAV1 that was coimmunoprecipitated with LATS1 (Fig. 4*B* and Fig. S5*D*). Taken together, these data suggested that CORO7 acts as a scaffold for LATS1, helping it to interact with SAV1 and MST2. This result was further corroborated by observing a decreased binding of endogenous LATS1 and SAV1 to overexpressed MST2 on *CORO7* knockdown (Fig. 4*C* and Fig. S5*E*). Surprisingly, the depletion of *CORO7* reduced the binding of SAV1 to MST2 (Fig. 4, *A* and *C* and Fig. S5, *B*, *C*, and *E*), suggesting a role of CORO7 in formation or stabilization of the SAV1–MST1/2 complex. Collectively, we concluded that CORO7 is necessary for the formation of Hippo core kinase complex.

### The interaction between CORO7 and SAV1 decreases on activation of the Hippo pathway

**Figure 5:**
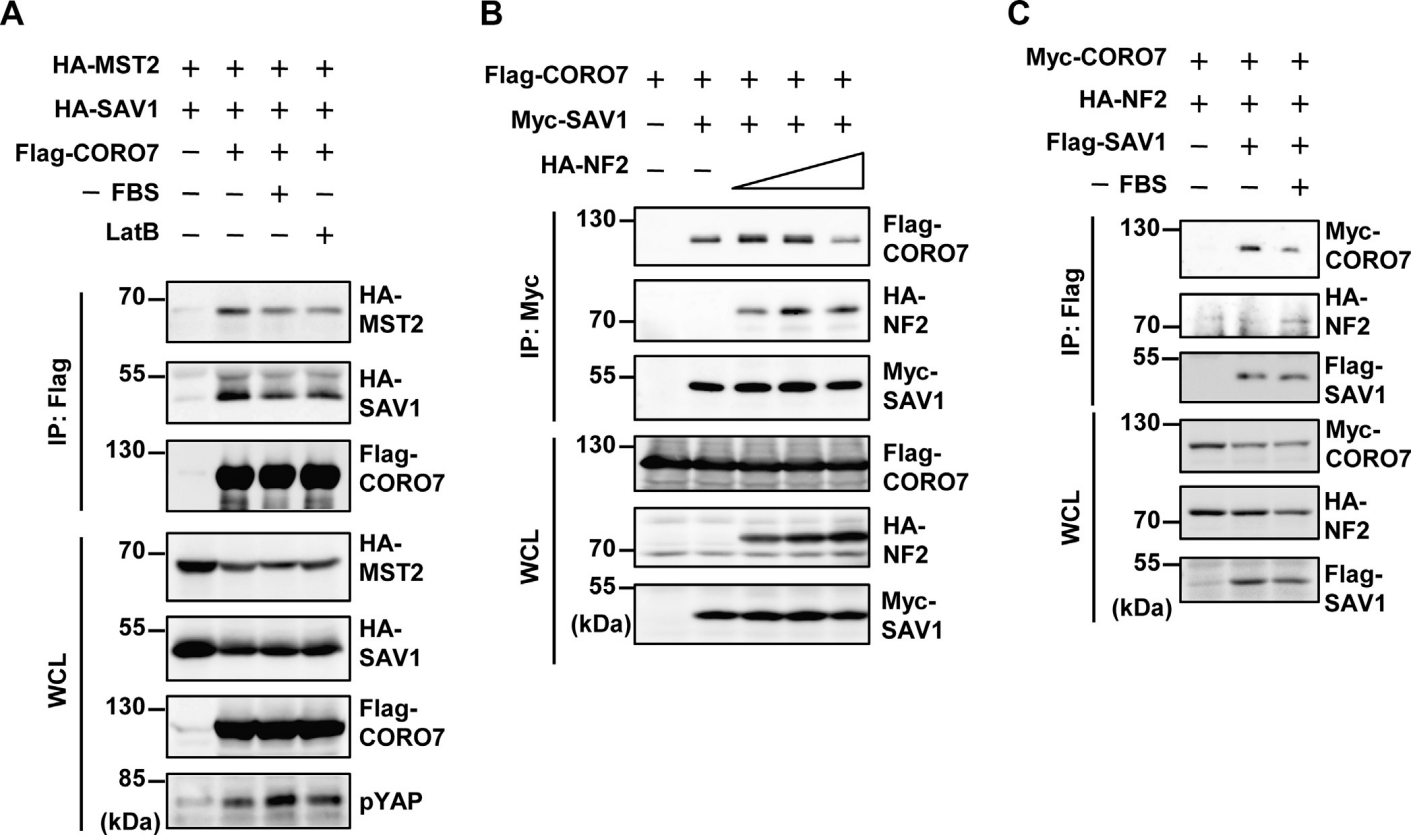
**Signals activating the Hippo pathway regulate complex formation between CORO7 and the components of the pathway.***A,* 24 h after seeded to 15% confluency, HEK293T cells transfected with HA-MST2, HA-SAV1, and Flag-CORO7 were deprived of serum (FBS) or treated with 0.25 μg/ml LatB for 1 h. The cell lysate samples were immunoprecipitated with anti-Flag antibody and immunoblotted with anti-HA and anti-Flag antibodies. The WCL samples were loaded for indicating the expression levels. The WCL samples were also immunoblotted with anti-pYAP antibody. *B,* HEK293T cells were transfected with Flag-CORO7, Myc-SAV1, and HA-NF2. Three different levels of HA-NF2 were expressed. The cell lysate samples were immunoprecipitated with anti-Myc antibody and immunoblotted with anti-HA, anti-Flag, and anti-Myc antibodies. The WCL samples were loaded for indicating the expression levels. *C,* HEK293T cells expressing Myc-CORO7, HA-NF2, and Flag-SAV1 were deprived of serum (FBS). The cell lysate samples were immunoprecipitated with anti-Flag antibody and immunoblotted with the same antibodies as in (*B*). The WCL samples were loaded for indicating the expression levels. All immunoblots are representative of at least three independent experiments. CORO7, coronin 7; FBS, fetal bovine serum; HA, hemagglutinin; LatB, latrunculin B; MST2, mammalian sterile 20-like kinase 2; NF2, neurofibromatosis type II; SAV1, salvador family WW domain–containing protein 1; WCL, whole cell lysate; YAP, yes-associated protein.

As mentioned previously, serum starvation and actin depolymerization are two well-established signals activating the Hippo pathway in mammalian cells, and CORO7 was found to be crucial for such activation (Fig. 2). Therefore, we investigated whether these stimuli regulate the interaction of CORO7 with SAV1, MST2, and LATS1. Interestingly, when the Hippo pathway was activated by serum starvation or LatB-induced actin depolymerization, levels of SAV1 and MST2 that were coimmunoprecipitated with CORO7 decreased significantly (Fig. 5*A* and Fig. S6, *A* and *C*), whereas LATS1–CORO7 binding was not affected by the serum deprivation (Fig. S6*B*).

Previous studies have reported that SAV1 physically interacts with NF2 (45, 47), and coexpression of SAV1 and NF2 enhances the phosphorylation of LATS1/2 at its hydrophobic motif (22). Pan *et al.* suggested that the FBM of SAV1, which is responsible for SAV1–NF2 binding, is necessary for the role of SAV1 promoting this phosphorylation, presumably because the interaction of SAV1 and NF2 facilitates Hippo pathway activation. Considering that the CORO7-binding site on SAV1 is near the FBM where NF2 binds to (Fig. 3*C*), we hypothesized that NF2 would be involved in the alteration of interaction between CORO7 and SAV1. Indeed, the binding between SAV1 and CORO7 was reduced along with the expression of NF2 in a dose-dependent fashion (Fig. 5*B* and Fig. S6*D*). Next, we explored the possibility that the competition between CORO7 and NF2 for the interaction with SAV1 is affected by the activity of the Hippo pathway. Interestingly, SAV1 preferred to bind to NF2 over CORO7 after Hippo pathway activation induced by serum starvation (Fig. 5*C* and Fig. S6*E*). Collectively, these results suggested a possibility that CORO7 may affect the interaction between SAV1 and NF2 by being released from SAV1 on Hippo-activating signals.

### Tyrosine kinase Src is a potential regulator of CORO7

We then searched for mediators that link exogenous growth signals to mammalian CORO7 or *Drosophila* Pod1. With the same scheme used to find *pod1*, we listed genes that are referenced to interact with *CORO7* or *pod1* from interactome databases (Table S2) and tested their genetic interactions with *pod1* in *Drosophila*.

**Figure 6:**
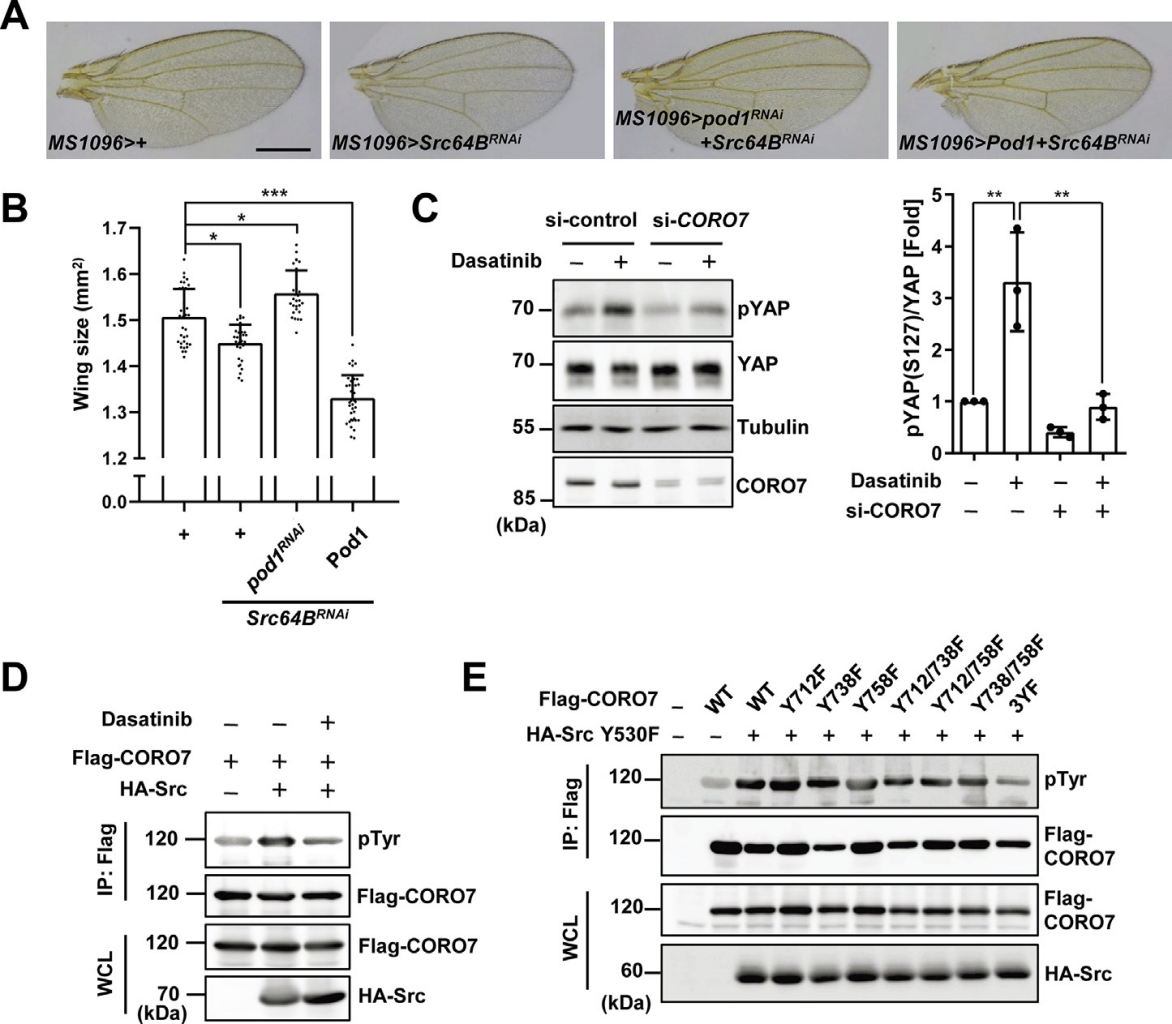
**Tyrosine kinase Src is a potential regulator of CORO7.***A,* representative pictures of the wings expressing RNAi targeting *Src64B* (*Src64B*^*RNAi*^) with or without expressing Pod1 or RNAi targeting *pod1* (*pod1*^*RNAi*^) by *MS1096*-GAL4 as indicated. The scale bar represents 500 μm. *B,* statistical analysis of the wing size of each genotype in (*A*). The error bars represent ±S.D. from n > 20. One-way ANOVA and Tukey post-test were applied (∗*p* < 0.05; ∗∗∗*p* < 0.001). *C,* siRNA targeting *CORO7* (si-*CORO7*) or nontargeting control siRNA (si-control) was transfected in MDA-MB-231 cells, and 50 nM dasatinib was treated for 1 h as indicated. The cell lysate samples were immunoblotted with anti-pYAP, anti-YAP, antitubulin, and anti-CORO7 antibodies. Quantification of the fold ratio of pS127-YAP bands to YAP protein levels under each indicated condition (*right*). The error bars represent ±S.D. from n = 3. One-way ANOVA and Tukey post-test were applied (∗∗*p* < 0.01). *D,* HEK293T cells expressing Flag-CORO7 and HA-Src were treated with 250 nM dasatinib for 2 h. The cell lysate samples were immunoprecipitated with anti-Flag antibody and immunoblotted with anti-pTyr and anti-Flag antibodies. The WCL samples were loaded for indicating the expression levels. *E,* HEK293T cells were transfected with indicated mutant forms of Flag-CORO7 and a constitutively active form of Src (HA-Src Y530F). CORO7 3YF indicates that Tyr712, Tyr738, and Tyr758 were all mutated to phenylalanine. The lysate samples were immunoprecipitated with anti-Flag antibody and immunoblotted with anti-pTyr and anti-Flag antibodies. The WCL samples were loaded for indicating the expression levels. CORO7, coronin 7; YAP, yes-associated protein; HA, hemagglutinin; WCL, whole cell lysate.

Among the 13 candidates, *Src* has already been documented to regulate the Hippo pathway in previous studies (48, 49, 50, 51). Therefore, we investigated whether *Src64B*, the *Drosophila Src* ortholog, interacts with *pod1* to regulate the Hippo pathway. As a result, similar to the overexpression of Pod1, the knockdown of *Src64B* led to a smaller wing phenotype, which was completely restored on collateral knockdown of *pod1* (Fig. 6, *A* and *B*; see also Fig. 1, *B* and *C*). Importantly, as knockdown of *pod1* in wings did not result in larger wings, we excluded the possibility of an additive effect where silencing *pod1* affected the phenotype independently to *Src64B*. Furthermore, overexpressing Pod1, which caused smaller wing sizes compared with wild-type *Drosophila*, consistently resulted in decreased wing sizes of *Src64B* knockdown mutants (Fig. 6, *A* and *B*). This genetic epistasis suggests *pod1* as a downstream target of *Src64B* in *Drosophila*.

To investigate whether Src plays a role in the Hippo pathway with regard to CORO7 in mammalian systems, we tested the effect of CORO7 manipulation on the pathway on pharmacological inhibition of Src. As a result, YAP Ser127 phosphorylation increased on the treatment of dasatinib, a Src inhibitor, whereas *CORO7* depletion decreased the YAP phosphorylation in dasatinib-treated cells (Fig. 6*C*), suggesting that CORO7 is required for Src to regulate the Hippo pathway. Furthermore, overexpression of wild-type Src increased tyrosine phosphorylation of CORO7, and treatment with dasatinib attenuated the level of this phosphorylation (Fig. 6*D*). Based on these results, we hypothesized that Src inhibits the Hippo pathway through phosphorylating CORO7 on tyrosine residues. As we assumed that the regulation of CORO7 by Src might be conserved in mammalian systems and *Drosophila* according to our experimental results described so far, seven tyrosine residues that are conserved in both human and *Drosophila* were tested whether those sites can be phosphorylated by Src. As a result, combinatorial mutations of Tyr712, Tyr738, and Tyr758 of CORO7 (3YF) resulted in a significant decrease of tyrosine phosphorylation by a constitutively active Src (Src^Y530F^) (Fig. 6*E*). Of note, phosphorylation of CORO7 Tyr758 by Src has been reported to be responsible for the association of CORO7 with the Golgi membrane (34). These data collectively implicate a possible role of Src in regulating CORO7.

## Discussion

In the present study, we identified CORO7 as a positive regulator of the Hippo pathway. After finding genetic interactions between *pod1* and Hippo pathway genes in *Drosophila*, we demonstrated that its ortholog *CORO7* is required for the activation of the pathway in mammalian cells. We showed that overexpressing Pod1 suppresses the phenotypes induced by knocking down *Hpo* or overexpressing Yki in wings and eyes of *Drosophila*, whereas YAP inhibition induced by various stimuli activating the Hippo pathway is repressed on *CORO7* depletion in mammalian cells. CORO7, which has two conserved WD40-repeat domains, interacts with SAV1, MST2, and LATS1, which are three major components of the Hippo pathway. We observed that this double WD40-repeat protein, whose upstream regulator may be a tyrosine kinase Src according to our experiments, is necessary for the formation of the Hippo core kinase complex, suggesting its function as a critical scaffold protein in the Hippo pathway.

Although WD40-repeat domains are well known for their roles in facilitating protein–protein interactions, there has been no evidence that CORO7 works as a scaffold. In the present study, we showed the interaction of CORO7 with three different components of the Hippo core kinase complex as well as its role in the formation of the complex (Figs. 2 and 3). Interestingly, the interaction between CORO7 and the SAV1–MST1/2 complex was rather decreased in response to Hippo-activating signals (Fig. 5). Based on our coimmunoprecipitation data, it is possible that CORO7 competes with NF2 for binding to SAV1 with altered affinity depending on the activity of the Hippo pathway, which could compromise the positive role of CORO7 considering that NF2 is an essential component in the Hippo pathway. Therefore, we suggest that CORO7 may help to form the core kinase complex in the first stage and then be released from the complex to let NF2 interact with SAV1 in the second stage of activation (Fig. S7). This idea could extend the previous report regarding the role of SAV1–NF2 interaction in the Hippo pathway (22).

In addition to its scaffolding function, CORO7 may affect LATS1 stability on Hippo-activating signals. Our data showed that when *CORO7* is depleted, the protein level of LATS1 decreases under Hippo-activated conditions (Fig. S3, *C* and *D*). It has been documented that LATS1 stability control by E3 ubiquitin ligases, including itchy E3 ubiquitin protein ligase and neural precursor cell-expressed developmentally downregulated gene 4, is one of the key regulatory mechanisms of the Hippo pathway (52, 53, 54). Therefore, besides its function as a scaffold, CORO7 may play a protective role for LATS1 in terms of protein stability.

It has been reported that CORO7 is associated with actin cytoskeleton assembly (32, 33, 35), which itself is also closely related to the regulation of the Hippo pathway. Therefore, one can consider the possibility that the alteration of the Hippo pathway in *CORO7*-deficient cells or *pod1*-deficient *Drosophila* might result from the actin-related roles of CORO7. However, studies have shown that loss of CORO7 leads to depolymerization of the actin cytoskeleton, which would result in the activation of the Hippo pathway, contradicting the inhibitory effect of CORO7 deficiency on the pathway demonstrated in this study. Thus, although it would be interesting to further study the relationship among the actin cytoskeleton, CORO7, and the Hippo pathway, we concluded that modulation of the Hippo pathway by CORO7 may not occur mainly via its control of actin stabilization.

A few studies have recently reported the roles of Src in the regulation of the Hippo pathway. In mammalian systems, Src negatively regulates the Hippo pathway by directly phosphorylating LATS1, leading to its inhibition (49, 50). Other studies showed that YAP and transcriptional co-activator with PDZ-binding motif are also targets of phosphorylation by Src, which induces their increased activity (48, 55). These regulations of LATS and YAP depend on Src activation by adhesion of cells to the extracellular matrix, which has also been known to serve as an upstream signal inhibiting the Hippo pathway (50, 56). Moreover, in *Drosophila*, *Src64B* activates c-Jun N-terminal kinase signaling, consequently suppressing the Hippo pathway (51, 57). In addition to these previous studies, we propose yet another mechanism by which Src may regulate the Hippo pathway through CORO7 phosphorylation. Further studies will be needed to understand the relationship between Src and CORO7 in the Hippo pathway.

Taken together, we suggest CORO7 as an evolutionarily conserved member of the Hippo pathway, functioning as a scaffold that not only tethers the components altogether but also acts as a critical regulatory point. As it is of great importance to elucidate precise molecular mechanisms of the assembly and regulation of the Hippo core kinase complex for better comprehension of the pathway, our study is thought to add a piece of understanding by implying that CORO7 is a promising subject for future research pursuit.

## Experimental procedures

### Drosophila strains and genetics

All *Drosophila* strains were maintained at 25 °C. *w*^*1118*^ strains were used for wild-type control. Following *Drosophila* strains were used in this study: *GMR*-GAL4 (Bloomington Drosophila Stock Center [BDSC] 9146), *MS1096*-GAL4 (BDSC 8860), *heat shock*-GAL4 (BDSC BL2077), *Hedgehog*-GAL4 (BDSC 67046), UAS-eGFP (BDSC 5430), UAS-Pod1 (BDSC 8801), UAS-*pod1*^RNAi^ (VDRC 108886), UAS-*Hippo*^RNAi^ (from Dr Georg Halder), UAS-Yorkie:GFP and UAS-Yorkie^S168A^:GFP (from Dr Ken Irvine), UAS-Sd (BDSC 9374), and UAS-*Src64B*^RNAi^ (BDSC 62157).

### Wing mounting

*Drosophila* wings were mounted using Gary's Magic Mountant, a mixture of Canada balsam and methyl salicylate (4:1 v/v). Images of adult *Drosophila* wings were taken by Leica DM750 equipped with ICC50E (Leica) using 5× (numerical aperture: 0.15) chroma objectives. Data acquisition was performed using Leica Application Suite, version 4.10.0 (Leica).

### Generation of pod1 KO Drosophila by the CRISPR/Cas9 system

Following guide-RNA sequence was used: 5′CTTCGCCTCCGCCTTGGGCACAAT and 3′AAACATTGTGCCCAAGGCGGAGGC. Guide RNA was ligated into pU6 chi-RNA vector and injected with pBS-Hsp70-Cas9 into *w*^*1118*^ embryos.

### Drosophila imaging

Adult *Drosophila* imaging was performed using Stemi 2000-C (Zeiss) and Axiovision Rel 4.8 (Zeiss).

### Heat shock–induced gene expression in Drosophila

For heat shock–induced gene expression using *heat shock*-GAL4*, Drosophila* was incubated at 30 °C for 24 h.

### Mammalian cell culture and transfection

HEK293T and MDA-MB-231 cells were maintained in DMEM containing 10% of fetal bovine serum. For serum deprivation and LatB (0.25 μg/ml) treatment and cell detachment experiments, cells were seeded at low confluency (less than 30%). HEK293T and MDA-MB-231 cells were treated with dasatinib (50 nM or 250 nM) for 1 h or 2 h, respectively (Sigma-Aldrich; CD S023389). Plasmids were transfected with the polyethylenimine reagent. HEK293T cells were provided by Dr John Blenis and MDA-MB-231 cells by Dr Dae-sik Lim. A nontargeting siRNA was used for siRNA control (Bioneer, 1003). *CORO7* siRNA (Bioneer, 5′-CACCTTGTGTCTACTGGAT-3′ and 5′-ATCCAGTAGACACAAGGTG-3′) was transfected to HEK293T cells and MDA-MB-231 cells using the RNAiMAX reagent (Invitrogen) according to the manufacturer's protocol.

### Plasmids

The mammalian expression plasmids for human LATS1/2, MST1/2, SAV1, and MOB1 were gifts from Dr Dae-sik Lim (KAIST, Korea). CORO7 plasmid was purchased from Sino Biological (Beijing) and cloned in pcDNA3-hemagglutinin (HA), pcDNA3.1-Flag, and pcDNA3-Myc vector. Plasmids for truncated forms of genes were generated by PCR and cloned in pcDNA3-HA or pcDNA3.1-Flag expression vectors. LATS1-truncated mutants were gifted from Dr Nam-Gyun Kim (University of Washington School of Medicine, Seattle). Src plasmid was purchased from Addgene and cloned into pcDNA3-HA vector. SAV1, CORO7, and Src mutants were generated by site-directed mutagenesis. Sequences of all constructs were confirmed by DNA sequencing.

### Immunostaining

For immunostaining in *Drosophila*, wandering larvae were dissected in PBS and then fixed with 4% paraformaldehyde. Samples were washed two times with PBS with 0.1% Triton X-100 (PBST), permeabilized with 0.5% PBST, again washed with 0.1% PBST, and incubated with 3% bovine serum albumin in 0.1% PBST. The primary antibody was treated with the noted ratios for overnight at 4 °C. On the next day, the primary antibody solution was removed, and samples were washed by 0.1% PBST three times and incubated 2 h at room temperature with the tetramethylrhodamine- or 647-conjugated secondary antibodies (Jackson, 1:200; Invitrogen, 1:200). The tissues were washed in 0.1% PBST three times and then washed in PBS twice shortly and mounted on a glass slide with 80% PBS + glycerol solution. Images were taken using the Zeiss 10 program. Following antibodies and chemical were used: disc-large (4F3 from Developmental Studies Hybridoma Bank [DSHB], 1:200), DE-cadherin (DCAD2 from DSHB; 1:200), and Hoechst 33258 (Invitrogen, 1:400). For immunostaining in mammalian cells, cells seeded and grown on coverslips were fixed with 4% paraformaldehyde for 10 min at room temperature and then permeabilized with 0.1% Triton X-100 containing PBS. After blocking in goat serum for 1 h, slides were incubated with primary antibody for 1 h at room temperature or 4 °C overnight, washed three times with PBS, and then incubated with FITC- or tetramethylrhodamine-conjugated secondary antibodies (Jackson; 1:100) and Hoechst 33342 (Invitrogen; 1:1,000) for 1 h at room temperature. A primary antibody against YAP (sc-101199 from Santa-Cruz, 1:100) was used. The slides were then washed three times with PBS and mounted. Epifluorescence images were taken by LSM 710 (Zeiss) equipped with LSM T-PMT (Zeiss) at 23 °C using 20× (NA: 0.8) and 40× (NA: 1.20) chroma objectives. Data acquisition was performed using ZEN 2010 (Zeiss).

### Immunoblotting and immunoprecipitation

Cells were lysed in lysis buffer (150 mM NaCl, 20 mM Tris, pH 7.4, 1 mM EGTA, 1 mM EDTA, 1% NP-40, 2.5 mM sodium pyrophosphate, 1 mM Na_3_VO_4_, 10% glycerol, and protease inhibitors). Equivalent protein quantities were subjected to SDS-PAGE and transferred to nitrocellulose membranes. Membranes were blocked with 5% bovine serum albumin and 0.1% Tween-20 containing TBS for 1 h at room temperature and then incubated with the indicated primary antibodies at 4 °C overnight, followed by the appropriate HRP-conjugated anti-mouse/anti-rabbit secondary antibodies (Jackson) at room temperature for 1 h. Immunoreactive bands were visualized with enhanced chemiluminescence. Relative signal intensities of immunoblots were analyzed using Image Studio Lite 5.2 software (LI-COR Biosciences). For immunoprecipitation, cells grown in 21.5 cm^2^ culture plates were collected and lysed in 0.5 ml lysis buffer–containing protease inhibitors for 30 min at 4 °C. After 12,000*g* centrifugation for 15 min, the lysates were immunoprecipitated with 2 μg of specific antibody overnight at 4 °C, and 30 μl protein A/G agarose beads were washed and then added for an additional 1 h. Thereafter, the precipitants were washed five times with lysis buffer, the immune complexes were boiled with loading buffer for 5 min, and analyzed by SDS-PAGE. The following antibodies were used for immunoblotting and immunoprecipitation: antibodies against HA (MBL Life Science; M132-3), Flag (MBL Life Science; M185-3L), Flag (CST; 2368), Myc (MBL Life Science; M192-3), CORO7 (Abcam; ab117446), YAP (Santa-Cruz; sc-101199), p-YAP (CST; 4911), LATS1 (CST; 3477), tubulin (DSHB; E7), SAV1 (CST; 13301), p-Tyr (CST; 9411), and IgG (Millipore; 2896738).

### RNA isolation and real-time PCR

Total RNA was isolated from *Drosophila* and cultured cells using Trizol reagent. Complementary DNA was synthesized by reverse transcription using oligo (dT) and subjected to real-time PCR with *CTGF*, *CYR61*, and *ACTB* primers in the presence of SYBR green PCR-Mix (Bioneer, Korea). The relative abundance of *diap1* mRNA was calculated by normalization to *rp49* mRNA. The following primer pairs were used: *diap1* (5′-GCCACCGTATCGATATAGAGC-3′ and 5′-CCAACGACTCGACGCTGG-3′); *rp49*: (5′-GCTTCAACATGACCATCCGCCC-3′ and 5′-GCGCTTCTTGGAGGAGACGCCG-3′). The relative abundance of *CYR61* and *CTGF* mRNA was calculated by normalization to *ACTB* mRNA. The following primer pairs were used: *CYR61* (5′-GGTCAAAGTTACCGGGCAGT-3′ and 5′-GGAGGCATCGAATCCCAGC); *CTGF* (5′-ACCGACTGGAAGACACGTTTG-3′ and 5′-CCAGGTCAGCTTCGCAAGG-3′); *ACTB* (5′-CATGTACGTTGCTATCCAGGC-3′ and 5′-CTCCTTAATGTCACGCACGAT-3′). Data were obtained from five independent experiments and shown as the average mean ± S.D.

### CORO7 KO cell line establishment

CRISPR/Cas9 genome editing technology was used for the deletion of *CORO7*. The guide RNA sequences were cloned into the plasmid PX459 (Addgene; 62988), and the plasmids were transfected into MDA-MB-231 cells. About 24 h after transfection, the cells underwent puromycin selection (1 μg/ml) for 2 days and then were sorted into 0.33 cm^2^ culture plates with one cell per well. The clones were screened by Western blot with anti-CORO7 antibody. The guide RNA sequence was 5′-CACCGGCTTCAGGGTGTCCAAGTTC-3′.

### Luciferase assay

MDA-MB-231 cells were cultured in 9.6 cm^2^ culture plates and transfected with 400 ng 8×TBS luciferase and 20 ng pRL-TK using Lipofectamine 3000. Reporter activities were measured using the Dual Luciferase Kit (Promega) according to the manufacturer's guides.

### Statistical analysis

All experiments were repeated at least three times, and all scatter graphs with bars were expressed as mean ± S.D. For wing size comparison in *Drosophila*, one-way ANOVA and Tukey post-test were used to determine the statistical significance. For mammalian cells, the Student's two-tailed *t* test was used. Prism 5 (Graphpad) was used for the statistical analyses.

## Data availability

This article contains Tables S1 and S2 and Figs. S1–S7. All representative data are contained within the article and the supporting information file.

## Conflict of interest

The authors declare that they have no conflicts of interest with the contents of this article.
