# Supplementary material for: CORO7 functions as a scaffold protein for the core kinase complex assembly of the Hippo pathway
Source: J Biol Chem. 2020 Nov 23;296:100040. doi: 10.1074/jbc.RA120.013297 (PMC7949047; doi:10.1074/jbc.RA120.013297)
Supplement: Tables S1 and S2 and Figs. S1–S7 [file mmc1.pdf]

Supporting Information

CORO7 functions as a scaffold protein for the core kinase complex assembly of the Hippo pathway

Jina Park[1,2,3], Kyoungho Jun[1,2,3], Yujin Choi[1,2,3], Eunju Yoon[1,2], Wonho Kim[2], Yoon-Gu Jang[1,2], and Jongkyeong Chung[1,2*]

[1]School of Biological Sciences, Seoul National University
[2]Institute of Molecular Biology and Genetics, Seoul National University, 1 Gwanak-ro, Gwanak-gu, Seoul 08826, Republic of Korea

[3]These authors contributed equally.

[*]Corresponding author: Jongkyeong Chung
Tel: +82 2 880 4399; E-mail: jkc@snu.ac.kr

Running Head: CORO7 is a novel Hippo pathway component

Keywords: CORO7, *Drosophila*, Hippo pathway, pod1, Src

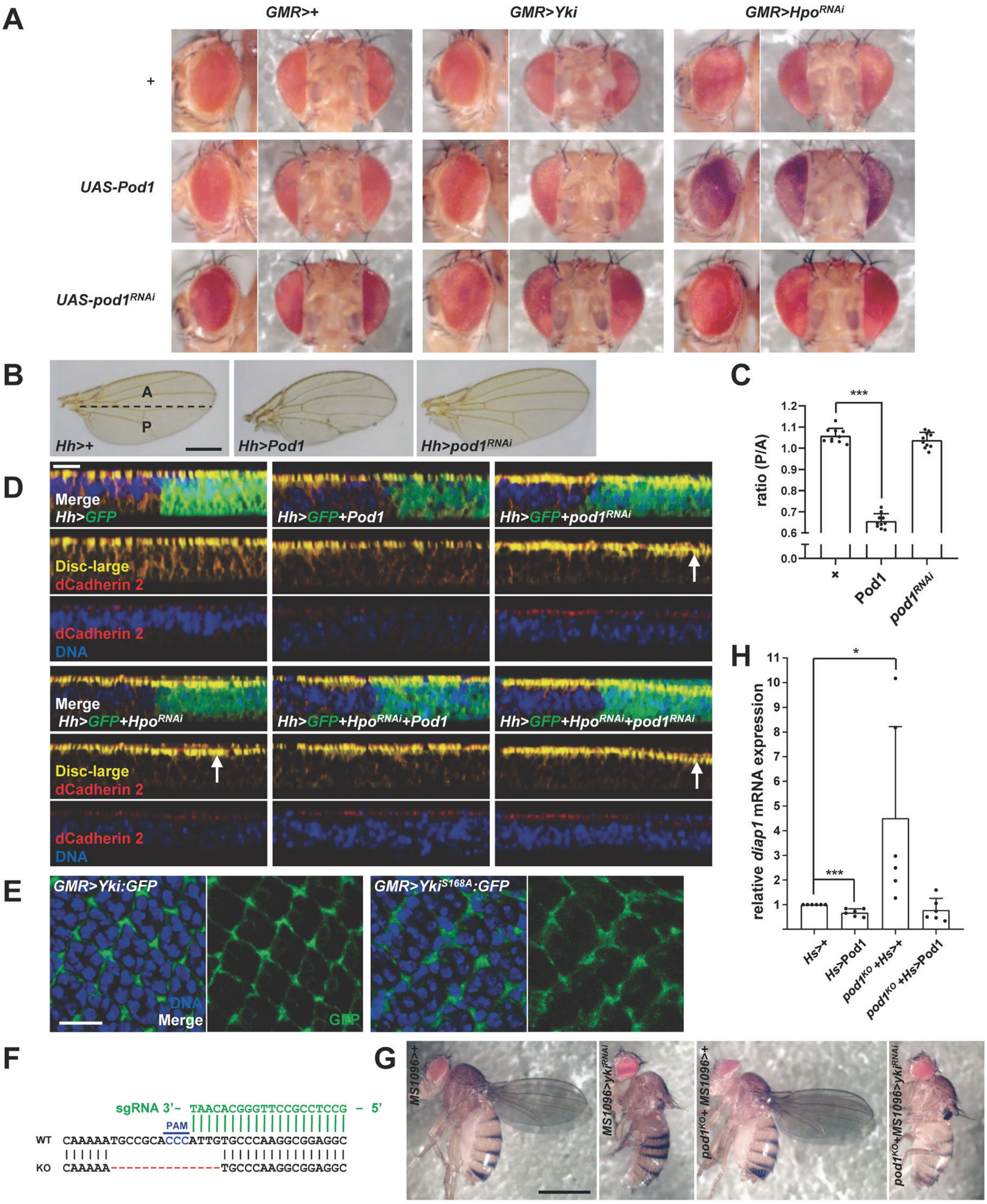
A
GMR>+
GMR>Yki
GMR>Hpo^RNAi
+
UAS-Pod1
UAS-pod1^RNAi
B
A
P
Hh>+
Hh>Pod1
Hh>pod1^RNAi
C
***
ratio (P/A)
1.2
1.1
1.0
0.9
0.8
0.7
0.6
0.0
+
Pod1
pod1^RNAi
D
Merge
Hh>GFP
Hh>GFP+Pod1
Hh>GFP+pod1^RNAi
Disc-large
dCadherin 2
dCadherin 2
DNA
Merge
Hh>GFP+Hpo^RNAi
Hh>GFP+Hpo^RNAi+Pod1
Hh>GFP+Hpo^RNAi+pod1^RNAi
Disc-large
dCadherin 2
dCadherin 2
DNA
E
GMR>Yki:GFP
GMR>Yki^S168A:GFP
DNA
Merge
GFP
F
sgRNA 3'- TAACACGGGTTCCGCCTCCG - 5'
PAM
WT CAAAAATGCCGCACCCATTGTGCCCAAGGCGGAGGC
KO CAAAAA-------------TGCCCAAGGCGGAGGC
G
MS1096>+
MS1096>yki^RNAi
pod1^KO+ MS1096>+
pod1^KO+MS1096>yki^RNAi
H
*
***
relative diap1 mRNA expression
11
10
9
8
7
6
5
4
3
2
1
Hs>+
Hs>Pod1
pod1^KO +Hs>+
pod1^KO +Hs>Pod1

Supplementary Figure 1. Genetic interaction between pod1 and Hippo pathway genes in adult *Drosophila.*

A. Pod1 overexpression or *pod1* knockdown was induced in eyes with or without the *Hpo* knockdown (*GMR>Hpo^{RNAi}*) or Yki:GFP overexpression (*GMR*>Yki) by *GMR*-GAL4. *B.* Pod1 overexpression or *pod1* knockdown was induced in the posterior (P) region of the wings by *Hh*-GAL4. Dashed line is indicated to emphasize the margins of anterior (A) and posterior (P) regions of the wing. Scale bar, 500 µm. *C.* Statistical analysis of the wing size of each genotype in (*B*). The ratio of the posterior (P) area over the anterior (A) area was measured. The error bars represent ± S.D. from n=10. One Way-ANOVA and TUKEY post-test were applied (***P<0.001). *D.* Vertical sections of *Drosophila* imaginal wing discs. The right half regions with GFP expression represent where GAL4-induced transgene is expressed. Pod1 overexpression (Pod1) or *pod1* knockdown (*pod1^{RNAi}*) was induced without or with *Hpo* knockdown (+*Hpo^{RNAi}*) by *Hh*-GAL4. Tissue overgrowth is indicated by arrows. Scale bar, 10 µm. *E.* Eye discs of *Drosophila* larvae expressing Yki:GFP or YkiS168A:GFP by *GMR*-GAL4. Scale bar, 10 µm. *F.* Genomic DNA sequences in wild-type (WT) or *pod1* KO *Drosophila* targeted by sgRNA. Protospacer adjacent motif (PAM) sequence is indicated. *G.* Representative pictures of *pod1* knockout (*pod1^{KO}*) *Drosophila* without or with *yki* knockdown (+*yki^{RNAi}*) by *MS1096*-GAL4. Scale bar, 1 mm. *H. diap1* mRNA expression levels of *pod1^{KO} Drosophila* without or with Pod1 overexpression (Pod1) by *heat shock*-GAL4 (*Hs*>) was determined by quantitative real-time PCR and normalized by a control gene, *rp49*. The error bars represent ± S.D. from 6 independent experiments. One Way-ANOVA and TUKEY post-test were applied (*P<0.05; ***P<0.001).

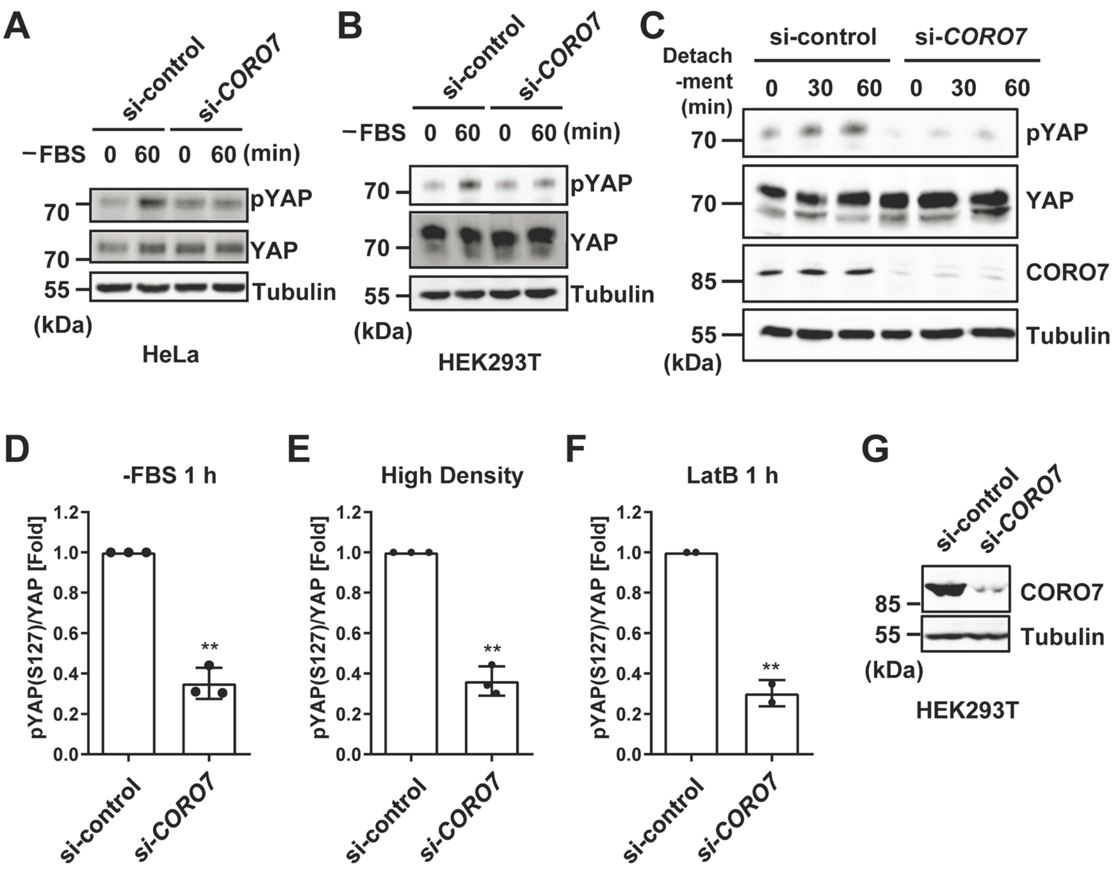

Supplementary Figure 2. CORO7 is critical for the activation of the Hippo pathway in mammalian cells.

A, B. HeLa (*A*) or HEK293 (*B*) cells were transfected with either non-targeting control siRNA (si-control) or siRNA targeting *CORO7* (si-*CORO7*) and were deprived of serum. The lysate samples were immunoblotted with anti-pYAP, -YAP, and -Tubulin antibodies. *C.* MDA-MB-231 cells were transfected with either non-targeting control siRNA (si-control) or siRNA targeting CORO7 (si-*CORO7*) and were treated with trypsin to detach from the plate. The lysate samples were immunoblotted with anti-pYAP, -YAP, -CORO7, and -Tubulin antibodies. *D-F.* Quantification of the ratio of pS127-YAP bands to YAP protein levels under each indicated condition for Fig. 2 A (*D*), 2 B (*E*), and 2 C (*F*). The error bars represent ± S.D. from n=2-3. Student's two-tailed t-test was applied (**P<0.01). *G.* Knockdown efficiency of siRNA for CORO7 used in Fig. 2 D and E was tested through immunoblotting for CORO7 and Tubulin.

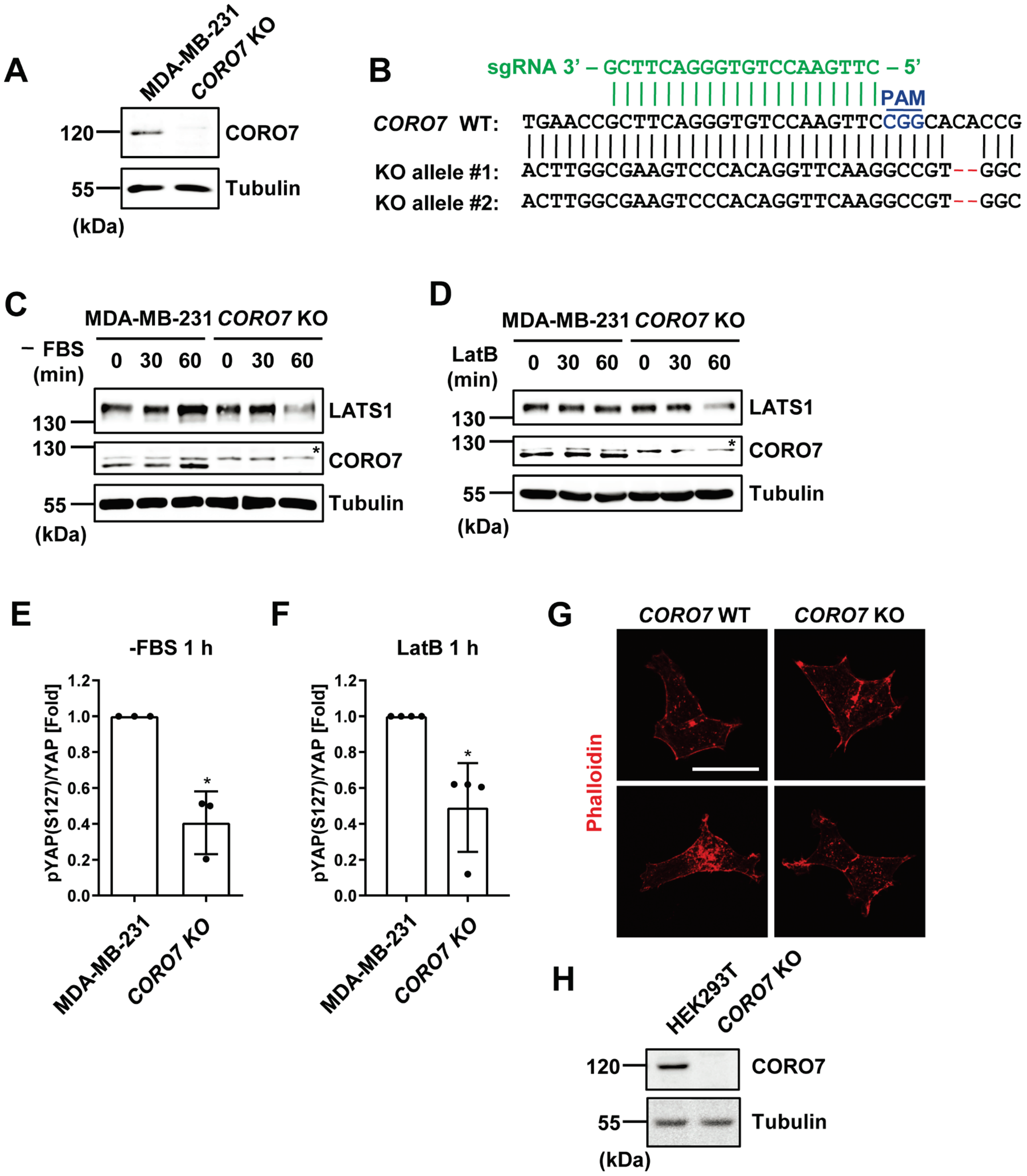

A
MDA-MB-231
CORO7 KO
120
CORO7
55
Tubulin
(kDa)
B
sgRNA 3' – GCTTCAGGGTGTCCAAGTTC – 5'
PAM
CORO7 WT: TGAACCGCTTCAGGGTGTCCAAGTTCCGGCACACCG
KO allele #1: ACTTGGCGAAGTCCCACAGGTTCAAGGCCGT--GGC
KO allele #2: ACTTGGCGAAGTCCCACAGGTTCAAGGCCGT--GGC
C
MDA-MB-231 CORO7 KO
− FBS (min)
0 30 60 0 30 60
LATS1
130
130
CORO7
55
Tubulin
(kDa)
D
MDA-MB-231 CORO7 KO
LatB (min)
0 30 60 0 30 60
LATS1
130
130
CORO7
55
Tubulin
(kDa)
E
-FBS 1 h
pYAP(S127)/YAP [Fold]
MDA-MB-231
CORO7 KO
F
LatB 1 h
pYAP(S127)/YAP [Fold]
MDA-MB-231
CORO7 KO
G
CORO7 WT
CORO7 KO
Phalloidin
H
HEK293T
CORO7 KO
120
CORO7
55
Tubulin
(kDa)

Supplementary Figure 3. Activation of the Hippo pathway is inhibited in CORO7 KO cells.

A. Wild-type (MDA-MB-231) or *CORO7* KO MDA-MB-231 cells were lysed and subjected to immunoblotting using anti-CORO7 and -Tubulin antibodies. *B.* Genomic DNA sequences in wild-type (WT) or *CORO7* KO MDA-MB-231 cells targeted by sgRNA. Protospacer adjacent motif (PAM) sequence is indicated. *C.* Wild-type MDA-MB-231 cells or MDA-MB-231 cells lacking *CORO7* (*CORO7* KO) were cultured under serum deprivation. The lysate samples were immunoblotted with anti-LATS1, -CORO7, and -Tubulin antibodies. *D.* Wild-type MDA-MB-231 cells or MDA-MB-231 cells lacking *CORO7* (*CORO7* KO) were treated with LatB (0.25 μg/ml). The lysate samples were immunoblotted with the same antibodies as in (*C*). As the cell lysate samples from Fig. 2 G were also used here for immunoblotting with anti-LATS1 antibody, the CORO7 and Tubulin blots in Fig. 2 G were shown in this panel again. Data information: Non-specific bands are indicated by asterisks in (*C*) and (*D*). *E and F.* Quantification of the ratios of pS127-YAP band intensities to YAP protein levels under each indicated condition for Fig. 2 F (*E*) and 2 G (*F*). The error bars represent ± S.D. from n=3-4. Student's two-tailed t-test was applied (*P<0.05). *G.* Actin was stained with phalloidin in CORO7 WT and KO HEK293T cells. Representative images are shown. Scale bar, 10 μm. *H.* Wild-type (HEK293T) or CORO7 KO HEK293T cells were lysed and subjected to immunoblotting using anti-CORO7 and -Tubulin antibodies.

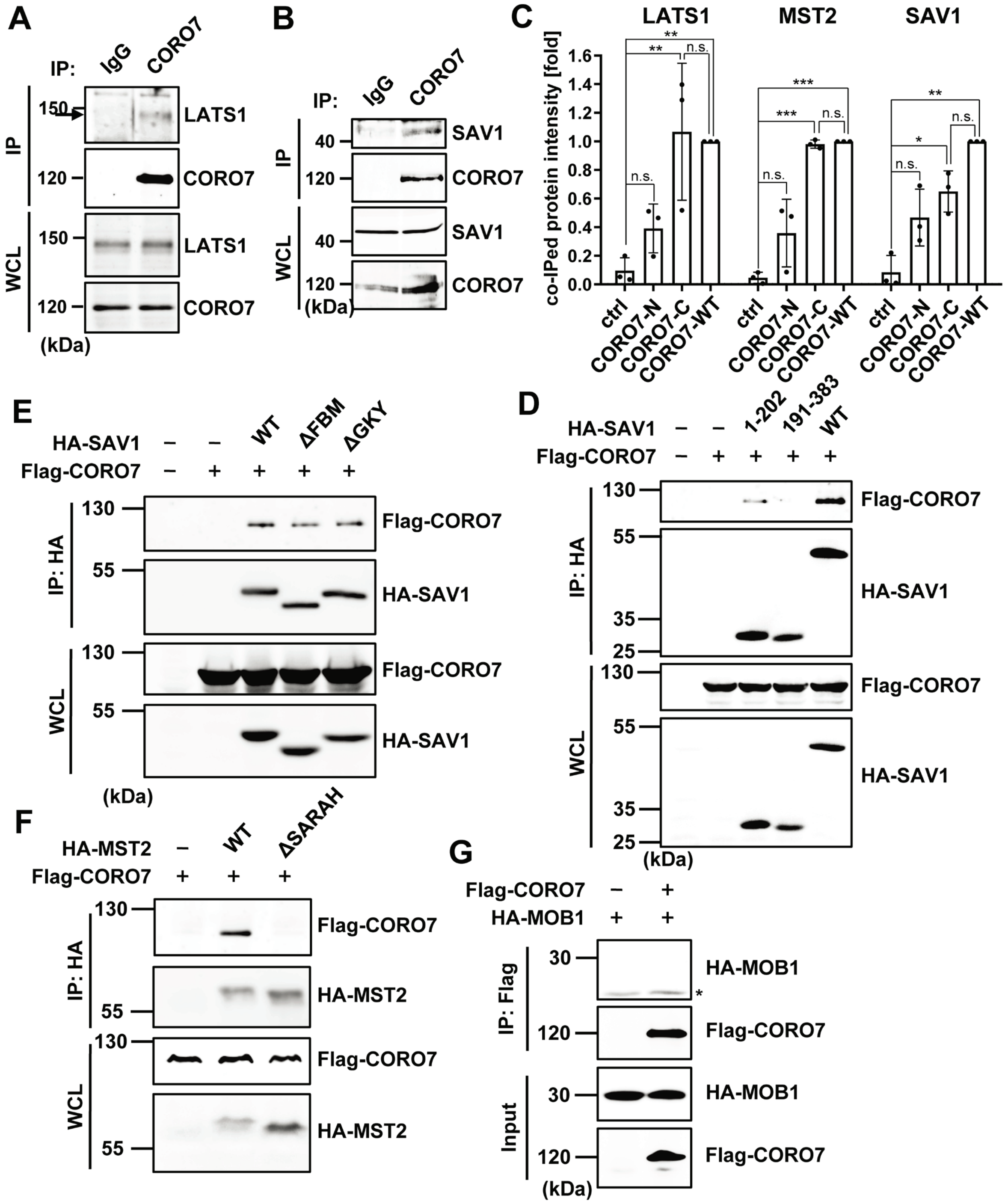

A
IP:
IgG
CORO7
150
LATS1
120
CORO7
IP
WCL
150
LATS1
120
CORO7
(kDa)
B
IP:
IgG
CORO7
40
SAV1
120
CORO7
IP
WCL
40
SAV1
120
CORO7
(kDa)
C
LATS1
MST2
SAV1
co-IPed protein intensity [fold]
n.s.
**
***
*
ctrl
CORO7-N
CORO7-C
CORO7-WT
D
HA-SAV1
1-202
191-383
WT
Flag-CORO7
IP: HA
WCL
Flag-CORO7
HA-SAV1
130
55
35
25
(kDa)
E
HA-SAV1
WT
ΔFBM
ΔGKY
Flag-CORO7
IP: HA
WCL
130
55
Flag-CORO7
HA-SAV1
(kDa)
F
HA-MST2
WT
ΔSARAH
Flag-CORO7
IP: HA
WCL
130
55
Flag-CORO7
HA-MST2
G
Flag-CORO7
HA-MOB1
IP: Flag
Input
30
120
HA-MOB1
Flag-CORO7
(kDa)

Supplementary Figure 4. CORO7 forms a complex with the components of the Hippo pathway.

A. Immunoprecipitation was performed on cell lysates from MDA-MB-231 cells with either anti-CORO7 or control (IgG) antibodies, and cell lysates were analyzed by western blotting for LATS1 and CORO7 level. *B.* Immunoprecipitation was performed on cell lysates from HEK293T cells with either anti-CORO7 or control (IgG) antibodies, and cell lysates were analyzed by western blotting for SAV1 and CORO7 level. *C.* Relative co-immunoprecipitated protein levels among vector, N-terminal, C-terminal or wild-type CORO7 with LATS1, MST2 or SAV1. Quantification for Fig. 3 D, 3 E, and 3 F. The error bars represent ± S.D. from n=3. One Way-ANOVA and TUKEY post-test were applied (n.s., non-significant; *P<0.05; **P<0.01; ***P<0.001). *D.* Flag-CORO7 was transfected with the HA-tagged truncated forms of SAV1 in HEK293T cells. The lysates were immunoprecipitated by anti-HA antibody and immunoblotted with anti-Flag and -HA antibodies. The whole cell lysate (WCL) samples were loaded for indicating the expression levels. *E.* Flag-CORO7 was transfected with the HA-tagged SAV1 wild-type or mutants (ΔFBM or ΔGKY) in HEK293T cells. The lysates were immunoprecipitated by anti-HA antibody and immunoblotted with the same antibodies as in (*D*). The whole cell lysate (WCL) samples were loaded for indicating the expression levels. SAV1 ΔGKY mutant lacks the first three amino acids (GKY) of the FBM in SAV1. *F.* Flag-CORO7 was transfected with the HA-tagged MST2 wild-type or mutant (ΔSARAH) in HEK293T cells. The lysates were immunoprecipitated by anti-HA antibody and immunoblotted with the same antibodies as in (*D*). The whole cell lysate (WCL) samples were loaded for indicating the expression levels. *G.* Flag-CORO7 was transfected with the HA-MOB1 in HEK293T cells. The lysates were immunoprecipitated by anti-Flag antibody and immunoblotted with the same antibodies as in (*D*). The whole cell lysate (WCL) samples were loaded for indicating the expression levels. Data information: Non-specific band is indicated by an asterisk in (*G*).

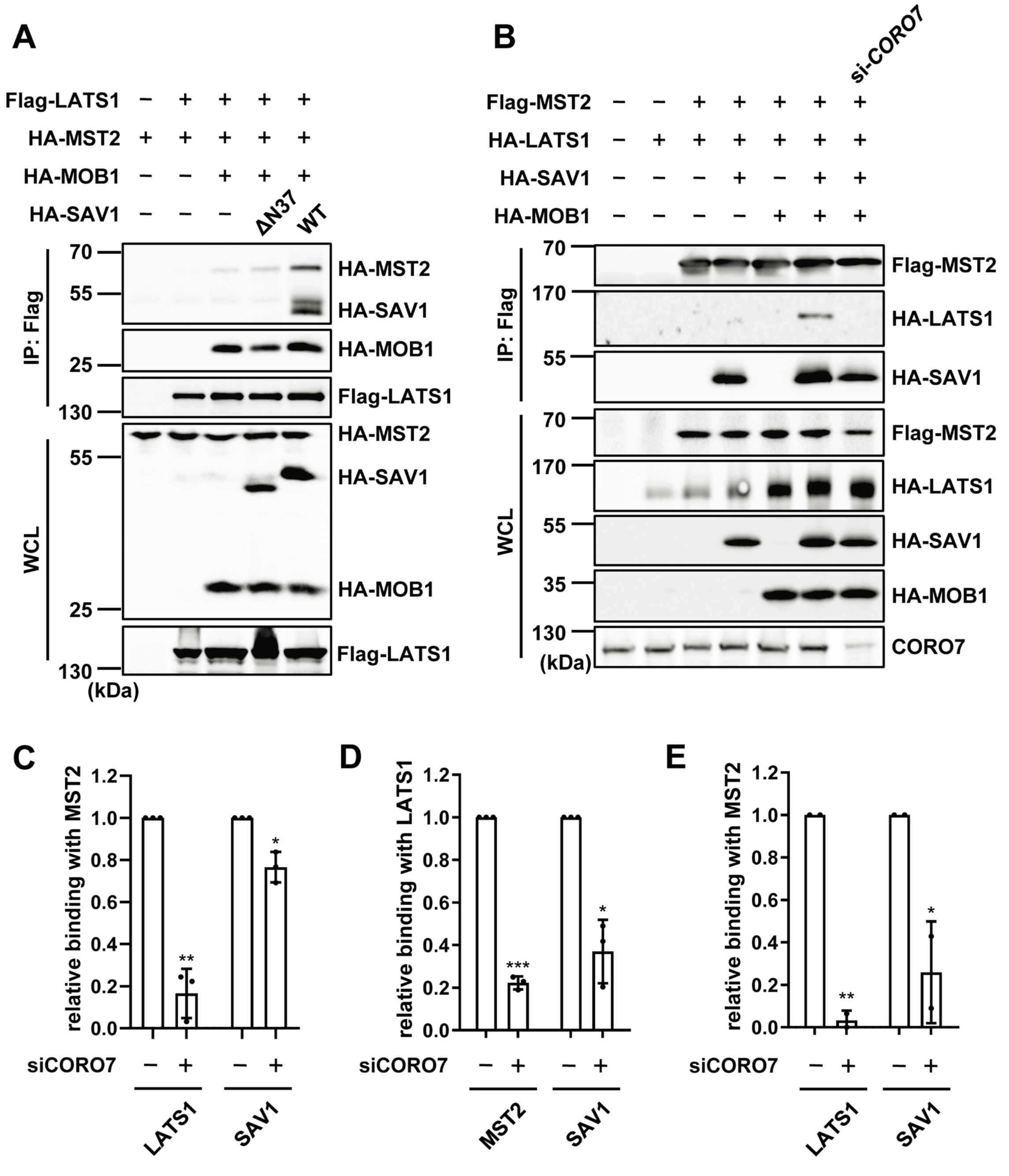

A
Flag-LATS1
HA-MST2
HA-MOB1
HA-SAV1
ΔN37
WT
IP: Flag
WCL
HA-MST2
HA-SAV1
HA-MOB1
Flag-LATS1
(kDa)
B
si-CORO7
Flag-MST2
HA-LATS1
HA-SAV1
HA-MOB1
CORO7
C
D
E
relative binding with MST2
relative binding with LATS1
siCORO7
LATS1
SAV1
MST2

Supplementary Figure 5. CORO7 is necessary for the formation of the core Hippo kinase complex.

A. HEK293T cells were transfected with Flag-LATS1, HA-MST2, HA-MOB1, and HA-SAV1 ΔN37 or wild-type. The lysates were immunoprecipitated by anti-Flag antibody and were immunoblotted with anti-HA and -Flag antibodies. The whole cell lysate (WCL) samples were loaded for indicating the expression levels. *B.* Flag-MST2, HA-LATS1, HA-SAV1, and HA-MOB1 were transfected in HEK293T cells to observe the co-immunoprecipitation between Flag-MST2 and the rest. siRNA targeting CORO7 was treated in the indicated lane (si-*CORO7*). The lysates were immunoprecipitated by anti-Flag antibody and were immunoblotted with anti-HA and -Flag antibodies. The whole cell lysate (WCL) samples were loaded for indicating the expression levels and CORO7 was immunoblotted to verify the efficiency of knockdown. *C.* Relative co-immunoprecipitated LATS1 or SAV1 with MST2 under each indicated condition. Quantification for Fig. 4 A. The error bars represent ± S.D. from n=3. Student's two-tailed t-test was applied (*P<0.05; **P<0.01). *D.* Relative co-immunoprecipitated MST2 or SAV1 with LATS1 under each indicated condition. Quantification for Fig. 4 B. The error bars represent ± S.D. from n=3 (*P<0.05; ***P<0.001). *E.* Relative co-immunoprecipitated LATS1 or SAV1 with MST2 under each indicated condition. Quantification for Fig. 4 C. The error bars represent ± S.D. from n=2. Student's two-tailed t-test was applied (*P<0.05; **P<0.01).

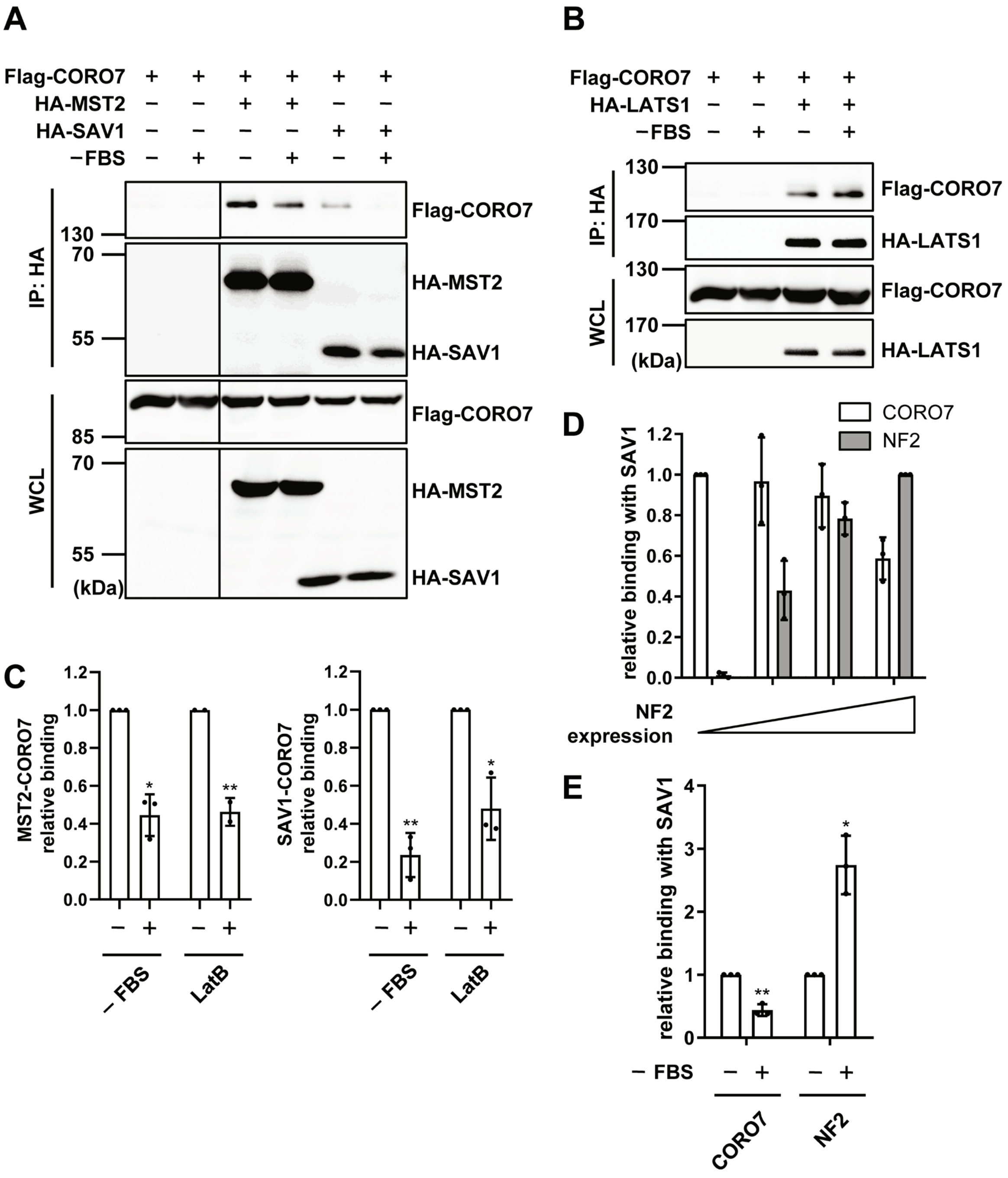

A
Flag-CORO7
HA-MST2
HA-SAV1
−FBS
IP: HA
WCL
130
70
55
85
(kDa)
Flag-CORO7
HA-MST2
HA-SAV1
B
HA-LATS1
170
C
MST2-CORO7 relative binding
SAV1-CORO7 relative binding
− FBS
LatB
D
CORO7
NF2
relative binding with SAV1
NF2 expression
E

Supplementary Figure 6. Regulation of the interaction of CORO7 with Hippo pathway components by Hippo-activating signals.

A. 24 h after seeded to 15% confluency, HEK293T cells transfected with Flag-CORO7 and HA-MST2 or HA-SAV1 were deprived of serum (-FBS) for 1 h. The cell lysate samples were immunoprecipitated with anti-HA antibody and were immunoblotted with anti-HA or -Flag antibodies. The whole cell lysate (WCL) samples were loaded for indicating the expression levels. *B.* 24 h after seeded to 15% confluency, HEK293T cells transfected with Flag-CORO7 and HA-LATS1 were deprived of serum (-FBS) for 1 h. The cell lysate samples were immunoprecipitated with anti-HA antibody and were immunoblotted with anti-HA or -Flag antibodies. The whole cell lysate (WCL) samples were loaded for indicating the expression levels. The two first lanes of (*A*) and (*B*) are identical. *C.* Relative co-immunoprecipitated MST2 or SAV1 with CORO7 under each indicated condition. Quantification for Fig. 5 A. The error bars represent ± S.D. from n=2-3. Student's two-tailed t-test was applied (*P<0.05; **P<0.01). *D.* Relative co-immunoprecipitated CORO7 or NF2 with SAV1 under each indicated condition. Quantification for Fig. 5 B. The error bars represent ± S.D. from n=3. *E.* Relative co-immunoprecipitated CORO7 or NF2 with SAV1 under each indicated condition. Quantification for Fig. 5 C. The error bars represent ± S.D. from n=3. Student's two-tailed t-test was applied (*P<0.05;**P<0.01).

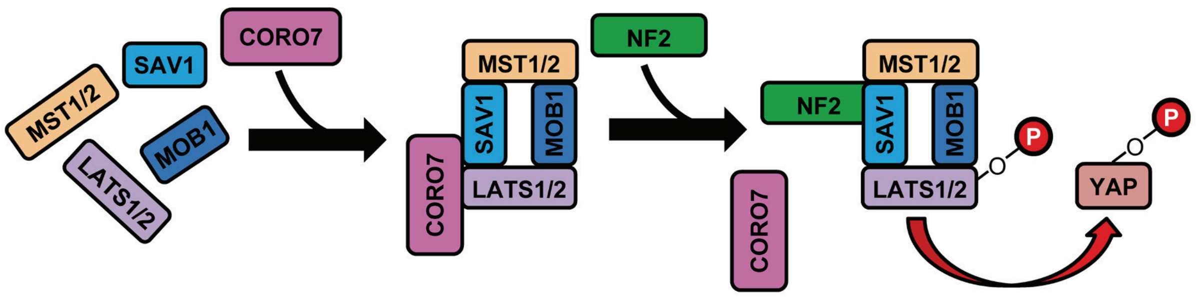

Supplementary Figure 7. Schematic model for the scaffold role of CORO7 in the Hippo pathway.

Supplementary Table 1. Candidate interactors for Hippo pathway genes from interactome-databases.

Gene	Interacting component	Gene	Interacting component	Gene	Interacting component
AGO1	Hpo	Fim	Ft, Mer	woc	Mer
Arf79F	Sav, Hpo	grn	Sav	ref(2)P	D, Mer
Ars2	Mer, Fj, Wts	HLH4C	Yki, Mer	Sec31	Ft, Yki
RnrS	Ft	Hrs	Ft, Fj	SEMG1	Sav
trn	Mer	igl	Mer	SERCA	Mts, Ds, Fj, Wts, Hpo
CG11999	Fj, Wts	inc	Ex	shrb	D, Mer
CG12645	Kibra, Sav	kune	Sav	SNCF	Ft, Mer
CG13705	Ft, Hpo	(1)G0289	Mts, Sav, Ds	Snx17	Ex
CG17841	Mer	Lasp	Ft, Yki	Spn	Tao, Mer
Cpsf5	Sav, Yki	Mbs	Dco, Mer	Tab2	Kibra, Yki
babo	Kibra	mod	Hpo, Mer	TER94	Ft, Mer
CG9581	Ds, Sav, Yki	mor	Sd, Yki, Mer	tum	D, Mer
dUfd2	Mer	mwh	Mer	Ythdf	Sav
chinmo	Hpo, Mer	NXPH2	FAT5	drk	Ex, Sav
Hel25E	Ft	pod1	D, Mer		

Supplementary Table 2. Candidate regulators for CORO7 from interactome-databases.

Gene	BioPlex	BioGRID	STRING	DroID
THBS3	O	O		
ASB6	O	O		
CNTF	O	O		
KLHL20	O	O	O	
SRC		O	O	
MYH10		O		O
RHOA		O		
AP1G1		O	O	
CUL1		O	O	
DAG1		O	O	
BUB1		O		O
NF2		O		O
BTBD10		O		O
